# Therapeutic Strategies for Dystrophin Replacement in Duchenne Muscular Dystrophy

**DOI:** 10.3389/fmed.2022.859930

**Published:** 2022-03-28

**Authors:** Cedric Happi Mbakam, Gabriel Lamothe, Jacques P. Tremblay

**Affiliations:** ^1^Centre de Recherche du CHU de Québec-Université Laval, Quebec City, QC, Canada; ^2^Department of Molecular Medicine, Faculty of Medicine, Laval University, Quebec City, QC, Canada

**Keywords:** artificial chromosome, progenitor cell, CRISPR/Cas 9, Duchenne muscular dystrophy, dystrophin, exon skipping, gene replacement, read through treatment

## Abstract

Duchenne muscular dystrophy (DMD) is an X-linked hereditary disease characterized by progressive muscle wasting due to modifications in the *DMD* gene (exon deletions, nonsense mutations, intra-exonic insertions or deletions, exon duplications, splice site defects, and deep intronic mutations) that result in a lack of functional dystrophin expression. Many therapeutic approaches have so far been attempted to induce dystrophin expression and improve the patient phenotype. In this manuscript, we describe the relevant updates for some therapeutic strategies for DMD aiming to restore dystrophin expression. We also present and analyze *in vitro* and *in vivo* ongoing experimental approaches to treat the disease.

## Introduction

Duchenne muscular dystrophy (DMD) is a lethal X-linked recessive disease caused by mutations in the *DMD* gene coding for dystrophin protein (1). The *DMD* gene has been found to be altered by more than 4,000 mutations leading to the absence of dystrophin expression under the sarcolemma of the affected patients (2). Nowadays, many therapeutic approaches to address the DMD pathology are elaborated with the final expectation of an effective and safe treatment to cure the disease. Some of these drugs have already obtained accelerated approval as treatments for DMD. However, so far none of these approved treatments or those still in ongoing clinical trials are able to restore a permanent expression of dystrophin under the sarcolemma. In the present review, we present the major molecular approaches for therapeutics leading to dystrophin restoration.

### Dystrophin Structure

**FIGURE 1:**
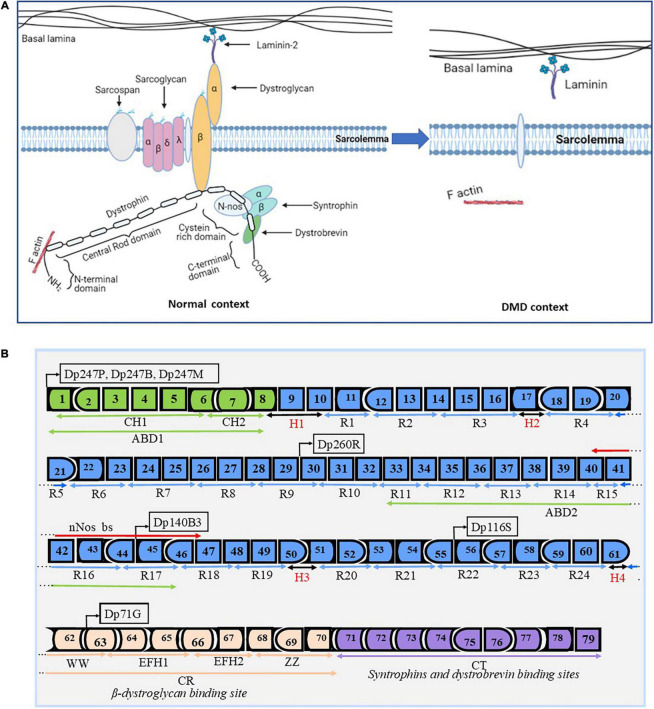
Dystrophin structure and protein associated domain. Design and adapted from the following references (1, 3, 147, 148). Panel **(A)** represents the basic structure of the dystrophin complex in the normal context and in a DMD context. In the normal context, dystrophin is present under the sarcolemma and is properly attached to other components to form the dystrophin complex. In the DMD context, there is an absence of the dystrophin complex, which results in the absence of the dystrophin complex and in muscle fiber degradation during muscle contraction. Panel **(B)** depicts the dystrophin gene (exons 1–79) with different functional domains: 1- the N-terminal actin binding domain (ABD1 in green) with its two calponin-homology motifs (CH1 and CH2). This domain is required for the function of the dystrophin protein. 2- The central rod domain (CR in blue color) contains 24 spectrin-like repeats (R1–R24), the normal alignment of this region is fundamental for a correctly functional dystrophin protein. This domain is interrupted and bordered by four proline rich hinge regions (H1, H2, H3, and H4). It also contains the second actin binding domain (ABD2) and the nNos binding site (nNos bs). 3- The Cysteine rich domain contains the WW domain, EF hands, and ZZ domain. This domain serves as the binding site for beta dystroglycan and it is required for a functional dystrophin. 4- The C terminal domain serves as a binding site for syntrophins and the dystrobrevin glycoprotein. This domain is also required for a functional dystrophin. The *DMD* gene encodes seven different protein isoforms under different tissues specific promoters (Dp427P, Dp427B, Dp427M, Dp260R, Dp140B3, Dp116S, and Dp71G, respectively for cerebellar Purkinje cells, brain, muscle, retina, and Schwann cells).

Dystrophin is one of the most important proteins required for structural and functional muscle dynamism (1). It is encoded by one of the biggest known genes in humans representing about 0.1% of the entire genome (3). The *DMD* gene contains 79 exons representing approximately 2,200 kb. The dystrophin protein is localized under the sarcolemma, the cell membrane of the striated muscle fibers (Figure 1A). This protein is divided into four essential domains. The first domain is the actin binding amino terminal domain (ABD1), which contains two calponin homology domains (CH1 and CH2) that bind directly to actin filaments. This binding links the dystrophin to the subsarcolemmal actin network and connects it to the contractile apparatus in skeletal muscle cells (4). The second domain is the dystrophin central rod domain, which contains 24 spectrin-like repeats, each made of three alpha helices. This domain contains the second actin binding motif (ABD2), localized around the middle of the rod domain, which interacts with the ABD1, forming a strong association with actin filaments. The rod domain interacts with microtubules and membrane phospholipids, it also provides elasticity to the dystrophin and facilitates interactions with other proteins through hinges made of proline rich spacers (3, 5). The third domain is the cysteine rich domain located between the rod domain and the carboxyl terminal domain. The cysteine rich domain binds the ankyrin, a protein that plays a key role in maintaining dystrophin under the sarcolemma. This domain is required for the dystrophin function (6). The fourth and last domain of dystrophin is the carboxy terminal domain, a crucial component of dystrophin that contains two regions forming alpha helical coiled coils, a protein motif involved as hinge protein into the protein–protein interaction. This domain also provides binding sites for syntrophin and dystrobrevin (3) (Figure 1B). These four domains interact with other structures of the cell cytoskeleton at the extra cellular level with alpha-dystroglycan, at the transmembrane level with dystroglycan, sarcoglycan, and sarcospan, and at the cytoplasmic level with dystrobrevin, syntrophin, and nNOS to form what is called the dystrophin complex (3). This complex is necessary for the maintenance of the membrane stability. Any modification in the dystrophin complex structure could result in various myopathies amongst which the DMD, which is the most common myopathy.

### Duchenne Muscular Dystrophy Pathogenesis

Duchenne muscular dystrophy is an inherited disease characterized by progressive muscle wasting due to mutations in the *DMD* gene coding for the dystrophin protein (7). There are many dystrophin isoforms whose expression generally depends on tissue-specific promoters, mRNA splice events, and poly-adenylation signal sites. The muscle dystrophin protein (Dp427m), a protein of 427 kDa, is the most important and the most implicated in DMD (8). The absence of dystrophin under the sarcolemma makes the muscle fibers more vulnerable during muscle contraction. Since it is a recessive X-linked disease, males are more likely to be affected (7).

The frequency of *DMD* mutations may depends on geography and race. In an updated systematic review and meta-analysis study conducted by Crisafulli et al. (9), the global prevalence of DMD was estimated to be 7.1 cases per 100,000 males and 2.8 cases per 100,000 individuals in the general population. In the pediatric population, the prevalence was about 19.8 per 100,000 live male births. In another study conducted by Ryder et al. (10), the DMD prevalence was evaluated at 10.9 per 100,000 males in France, 1.9 per 100,000 males in United States, 2.2 per 100,000 males in United Kingdom and 6.1 per 100,000 males in Canada.

Single or multi-exon deletions account in average for about 60–70% of cases depending on the geographical region of the world (64% in Oceania, 66% in Europe, 70% in America, 72% in Asia, and 88% in Africa) thus representing about 2/3 of all DMD cases (11). Two regions of the *DMD* gene are considered hotspots for mutations (12). The first and most important is between exons 45 and 55; deletions in this zone remove the central part of the rod domain. The second hotspot concerns the region between exon 3 to exon 19 of the *DMD* gene. Deletions in this zone remove all or part of the actin binding amino terminal domain as well as a section of the rod domain. Some exon deletions induce a frameshift mutation that results in a premature stop codon, which alters the dystrophin protein synthesis through the translational process. In a study conducted by Bladen et al. (11), some exons were found to be the most frequently mutated in DMD patients. Exon 51 represented about 14% of total *DMD* mutations and 21% of exon deletions. It was followed by exon 53, which carried 10% of total *DMD* mutations and 15% of exon deletions. Exon 45 represented 9% of total *DMD* mutations and 13% of exon deletions. Exons 43 and 44 each represented 7% of total *DMD* mutations and 11% of exon deletions. Point mutations represented around 26% of DMD cases. These represent nonsense and missense mutations, splice site mutations, and mid intronic mutations. DMD mutations also include 10–15% exonic and intronic duplications and about 3% small indels (3, 11).

Children with DMD initially show weakness of proximal muscles, abnormal gait and calf muscle pseudo-hypertrophy by the time they are three to 5 years old. Scoliosis is generally observed by the age of 12, patients usually die before the age of 30 due to severe cardiac and respiratory complications (13, 14). *De novo* mutations in the *DMD* gene have been found to be associated with DMD pathogenesis and HLA polymorphism (15). The *DMD* gene is one of only a few genes that can tolerate the deletion of one or many exons and still be translated into dystrophin protein with reduced functionality. A favorable prognostic was observed in a 7 years follow-up study involving three Becker patients with in-frame deletions of exons 45–55 (16). In another case, a 61 years old man had 46% of his *DMD* gene deleted, however, this gene was still capable of synthesizing a functional truncated dystrophin (17). The tolerance of the *DMD* gene to deletions is extremely relevant in the context of certain gene therapies, which seek to remove one or many exons to restore the expression of an internally deleted dystrophin and improve the symptoms of the patients.

## Molecular Biology Approaches for Duchenne Muscular Dystrophy

There are many strategies that researchers are developing to improve the phenotype of DMD patients. In this review, we discuss molecular biology approaches for the treatment of DMD: exon skipping, read through non-sense mutations, Cas9 based modification of the *DMD* gene, delivery of micro-dystrophin or full-length dystrophin gene, progenitor cell modulation, artificial chromosome for dystrophin transfer, gene replacement, and skeletal muscle cell transplantation.

### Read Through Nonsense Mutations

**FIGURE 2:**
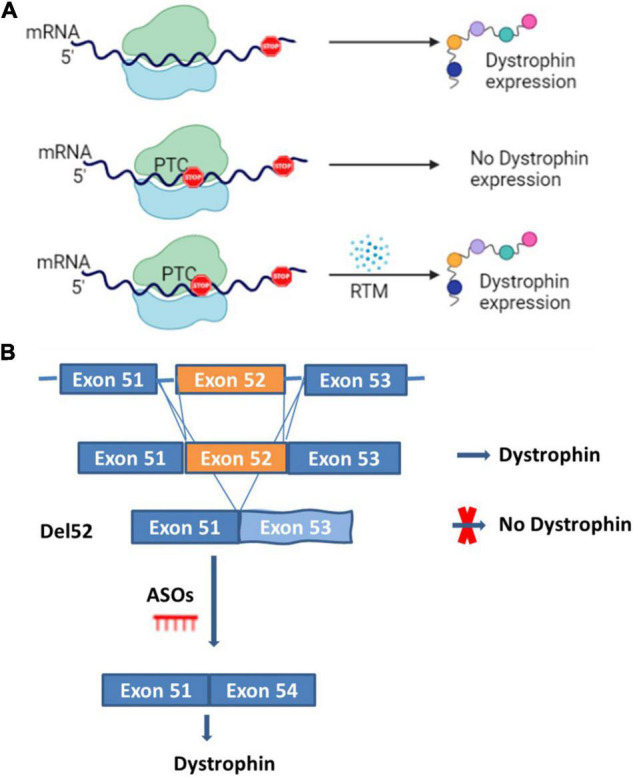
Read through and ASOs strategies. Panel **(A)** represents the normal dystrophin expression and a scenario where premature termination codon (PTC) is present and induces the absence of the dystrophin expression which can be restored by read through mutation molecules (RTM). Panel **(B)** represents a scenario for the DMD mutation amenable to exon 52 deletion (del52). In the presence of exon 52 there is dystrophin expression, in the case of exon 52 deletion, there is no dystrophin expression. Dystrophin expression can however be restored by inducing the skipping of exon 53 with anti-sense oligonucleotides (ASOs).

This approach targets stop codon point mutations that otherwise prevent the proper translation of the dystrophin mRNA (Figure 2A). Drugs are used to disrupt translational fidelity by interacting with the ribosomal RNA to translate the full length dystrophin despite the presence of the premature stop codon (18–20). Gentamicin was the first aminoglycoside drug used for this type of approach (21). A preliminary trial carried out with two DMD and two BMD patients, both possessing nonsense mutations, and treated once a day with gentamicin at 7.5 mg/kg/day for 2 weeks did not show any dystrophin expression (22). In a subsequent trial with four DMD patients carrying point mutations, a different protocol was used (two 6-day cycles of gentamicin sulfate, at an interval of 7 weeks) and the result revealed dystrophin expression in three out of four patients with a UGA mutation (23). In a cohort study carried out over 6 months with 12 DMD patients that had point mutations in different exons, six patients saw an increase in their levels of dystrophin after the treatment with six patients reaching real increased levels (post-treatment minus pre-treatment level of dystrophin) of 10.4, 10.9, and 11% respectively for point mutations in exons 25, 33, and 35 (18). There is no updated data about the Gentamicin potential for DMD treatment perhaps due to the lack of clear evidence of clinical efficacy as well as notable side effects such as hearing loss, kidney toxicity, injection site reactions, stomach upset, and neurotoxicity (24).

Other molecules such as Ataluren and Arbekacin sulfate (PTC Therapeutics Inc.) have also been reported to have some beneficial effects for DMD patients (20, 25). The beneficial results reported with Ataluren in a phase 2 clinical trial led to a phase 3 trial (26). Unfortunately, the phase 3 clinical trial results showed limited clinical benefits (27). In that trial carried out from 26 March 2013 to 26 August 2014, 230 participants were randomly divided into placebo and treated groups. There was no significant difference between Ataluren treated and placebo groups. Adverse effects were also reported for some patients (27). There is an ongoing phase 3 randomized, double-blind and placebo-controlled trial (NCT03179631) aiming to characterize the long-term effects of Ataluren-mediated dystrophin restoration in DMD patients. The study started in July 2017 and is expected to be completed by October 2023. In February 2021, a communication to the Duchenne community about preliminary results seemed to be encouraging (28).

### Treatment Using Exon Skipping

#### Antisense Oligonucleotide Approach

This approach uses antisense oligonucleotides (ASOs), which are designed to bind and modulate the RNA functions through different mechanisms (Figure 2B) (29). Here, the term oligonucleotide refers to a short, unmodified, or chemically modified nucleic acid. This sequence is theoretically capable of hybridizing to a complementary nucleic acid sequence (30). ASOs used in antisense therapeutics undergo different chemical modifications to improve their metabolic stability, binding specificity and affinity (30, 31). The major modifications include the sugar modifications (2′-*O*-methoxyethyl, 2′-*O*-methyl, 2′fluoro, locked nucleic acid and constrained ethyl bicyclic nucleic acid), the phosphate group modifications (methyl phosphonate, phosphorothioate, and phosphoramidate) and both the sugar and phosphate (morpholino) (29, 30). ASOs can act by the selective cleavage of RNA through the RNase H or the Ago 2, which is active in RNA-induced silencing complex (RISC). These molecules mediate the splice modulation by targeting splice junctions or exonic/intronic splicing enhancer/silencer sites resulting in the exon skipping (29, 30). The mechanisms for the removal of the exons are not completely clear. ASOs also mediate the regulation of microRNA involved in many diseases, the mRNA polyadenylation signals and the binding between proteins and pathogenic RNA (29, 30). ASO therapeutics for DMD restore the reading frame of the mRNA by excluding some of the exons during the maturation of the messenger RNA transcript. This resulting in a truncated dystrophin protein with reduce functionality (32, 33).

Since exon deletions represent about 60–70% of the total DMD cases, there are many ongoing clinical trials aiming to restore the normal reading frame of the dystrophin mRNA by skipping certain exons (Table 1). For example, trials are ongoing to skip exon 45 (Casimersen) (NCT03532542), exon 51 (Suvodirsen, Eteplirsen) (34), and exon 53 (Golodirsen, Viltolarsen) (35, 36).

**TABLE 1:** Updated summary of exon skipping strategy for DMD.

No	Drug	Study phase	Status	Period	Target	Trial number	Result available	Number of patients
1	–	–	Unknown	October 2011–December 2018	Exon 53 skipping	NCT01385917	No	45
2	Casimersen (SRP-4045)	1	Completed	October 2015–October 2018	Exon 45 skipping	NCT02530905	Yes	12
3	Casimersen/Eliptersen/Golodirsen	2	Enrolling	February 2020–September 2022	Exon 45/51/53 skipping	NCT04179409	No	6
4	Cesimersen-SRP-4045/Golodirsen-SRP-4053	3	Enrolling	August 2018–August 2026	Exon 45/53 skipping	NCT03532542	No	260
5	Cesimersen-SRP-4045/Golodirsen-SRP-4053	3	Recruiting	September 2016–May 2023	Exon 45/53 skipping	NCT02500381	No	222
6	DS-5141b	1 and 2	Completed	October 2015–October 2020	Exon 45 skipping	NCT02667483	No	7
7	DS-5141b	2	Enrolling	July 2020–March 2022	Exon 45 skipping	NCT04433234	No	8
8	Eteplirsen	3	Completed	November 2014–June 2017	Exon 51 skipping	NCT02255552	Yes	109
9	Eteplirsen	2	Completed	June 2015–December 2018	Exon 51 skipping	NCT02420379	Yes	33
10	Eteplirsen	2	Enrolling	June 2017–February 2027	Exon 51 skipping	NCT03985878	No	17
11	Eteplirsen	2	Completed	August 2017–March 2021	Exon 51 skipping	NCT03218995	No	15
12	Eteplirsen/EXONDYS 51	3	Active	July 2020–February 2026	Exon 51 skipping	NCT03992430	No	152
13	Eteplirsen-SRP-5051	2	Active	June 2019–May 2022	Exon 51 skipping	NCT04004065	No	70
14	Eteplirsen-SRP-5051	1 and 2	Active	December 2018–July 2024	Exon 51 skipping	NCT03675126	No	60
15	Golodirsen-SRP-4053	1 and 2	Completed	January 2015–March 2019	Exon 53 skipping	NCT02310906	Yes	39
16	PreU7-53	–	Unknown	October 2011–December 2018	Exon 53 skipping	NCT01385917	No	45
17	PRO-044	2	Completed	December 2014–August 2016	Exon 44 skipping	NCT02329769	No	15
18	Suvodirsen	2 and 3	Completed	September 2019–January 2020	Exon 51 skipping	NCT03907072	Yes	6
19	Viltolarsen	–	–	–	Exon 53 skipping	NCT04337112	No	–
20	Viltolarsen	3	Not yet recruiting	April 2021–June 2026	Exon 53 skipping	NCT04768062	No	74
21	Viltolarsen	4	Not yet recruiting	May 2021–November 2033	Exon 53 skipping	NCT04687020	No	16
22	Viltolarsen	3	Recruiting	April 2020–December 2024	Exon 53 skipping	NCT04060199	No	74

Eteplirsen also known as Exondys 51 (Sarepta Therapeutics, Inc.) was the first drug that obtained FDA accelerated approval. In DMD patients whose mutations are amenable to exon 51 skipping, this treatment has increased dystrophin expression by 0.28% and by 0.93% after 48 and 180 weeks of treatment respectively (34, 37). The phase 2 trial (NCT03218995), which evaluated the safety, efficacy and tolerability of Eteplirsen in 15 patients aged 6–48 months ended in March 2021, and the results published in clinical trial site indicated a serious adverse event (Bronchiolitis) in 1 patient aged less than 24 months. Some adverse events (not including serious) were also indicated in many patients. A long-term phase 3 clinical trial (NCT02255552) evaluated the efficacy of Eteplirsen in 18 patients ended in July 2019. A significant difference was demonstrated in the 6 min walk test (6MWT) between the baseline and 36 months post-treatment and six patients with a mean age of 9 lost ambulation (38). There is an ongoing phase 3 randomized, double-blind, dose finding and comparison study for the safety and efficacy of high dose of Eteplirsen. This study was preceded by the first phase of an open-label dose escalation study involving 144 DMD patients that is planned to be completed by February 2026. The next generation of Eteplirsen experimented with SRP-5051 that is ongoing clinical trials phase 1 and 2 (NCT03675126 and NCT04004065) is presented to have many advantages. The most relevant is the addition of cell penetrating peptides to the phosphorodiamidate morpholino oligomer (PMO) to form peptide phosphorodiamidate morpholino oligomer (PPMO), which leads to more efficient delivery, higher level of dystrophin production and efficient dosing for patients (39). Preliminary results from phase 2 MOMENTUM study revealed that SRP-5051 for DMD patients amenable exon 51 skipping dosed monthly at 30 mg/kg lead to mean dystrophin expression of 6.55% (39).

Viltolarsen or Viltepso™ (NS Pharma, Inc.) has also been granted accelerated approval by FDA in August 2020 to treat DMD patients amenable to exon 53 skipping (35, 40). Viltolarsen has shown in a phase 1/2 trial that the intravenous infusions of 80 mg/kg/week for 24 weeks increased the mean dystrophin level by 2.8% by western blot analyses (35). The final approval of this treatment will be determined by the outcome of the ongoing phase 3 randomized, double-blind, placebo-controlled, multi-center study (NCT04060199) to assess the efficacy and safety of Viltolarsen in ambulant boys with DMD amenable to exon 53 skipping. The study is planned to be completed by December 2024. In another phase 2 trial (NCT02740972) assessing the safety and intravenous dose of NS-065/NCNP-01 in DMD boys aged 4–9 years amenable exon 53 skipping, the authors showed a proof of beneficial functional and clinical effects in the group receiving low-dose (40 mg/kg per week) or high-dose (80 mg/kg per week) of Viltolarsen (40).

Golodersen also known as Vyondys 53 (Sarepta Therapeutics, Inc.) got an accelerated approval from the FDA in December 2019. Results from the phase 1/2 trials involving 12 participants showed a dystrophin mean level of 1.019% representing an approximate 16-fold increase in truncated dystrophin. A significant increase in dystrophin positive fibers was found at week 48 compared to the baseline level (36). The phase 3 trial (NCT02500381) to evaluate the effectiveness of Golodersen is still ongoing and is expected to be completed in May 2023. Another ongoing trial (NCT03532542) aiming to evaluate the long-term safety and tolerability is also expected to be concluded by August 2026.

Casimersen also known as AMONDYS 45™ (Sarepta Therapeutics, Inc.) got an accelerated approval from the FDA in February 2021 for the treatment of DMD patients amenable to exon 45 skipping (41). This conditional approval may be associated to the preliminary results obtained after 48 weeks of treatment for the ongoing trial (NCT02500381) where the mean dystrophin protein level in the treated group was 1.736% compared to 0.925% in the placebo group. There was also an increase in the mRNA level post-treatment compared to the baseline level (42). Side effects including upper respiratory tract infections, cough, fever, headache, joint pain, and pain in the mouth and throat were observed in about 20% of the patients. The study is estimated to be completed by May 2023. In the meantime, an ongoing long-term, open-label and extension trial (NCT03532542) in which 260 participants aged 7–23 years were enrolled is aiming to evaluate the long-term effects of AMONDYS 45™. The study is estimated to be completed by August 2026 and may determine the final approval of Casimersen.

### Small Nuclear RNA Approach

Another interesting approach for exon skipping is the use of small nuclear RNAs (snRNAs), which play a major role in mRNA splice modulation (43, 44). Decades ago, the snRNAs including U1snRNA and U7snRNA have been used as therapeutic approach targeting the dystrophin pre-messenger RNA to mediate skipping of exon 45 in human myoblasts (45) and exon 51 in mice (46) resulted in nearly complete restauration of dystrophin compared to wild type expression. This approach can be appropriately designed to mediate either a single or multi exon skipping (47). Recently, it has been shown that both the systemic and intramuscular injection of U7snRNA for the correction of *DMD* exon 2 duplication using a single complementary adeno-associated virus (scAAV) in a mouse model harboring exon 2 duplication (48), resulted in robust dystrophin expression and correction of muscle physiologic defects (49). In that study a dose-escalation experiment showed that the highest dose of scAAV U7 (7.6 × 10^13^ vg/kg) following a systemic injection in mouse tail vein resulted in 42–96% skipped transcripts (single or double skipping of exon 2) and 69–92% dystrophin positive fibers in skeletal muscle of 4 weeks post injection. Twelve weeks post injection permitted to achieve 85% positive fibers with progressive increase observed in absolute and specific force to finally reach complete rescue in absolute force and partial rescue of specific force. This improvement was also observed in heart muscle with up to 40% dystrophin level after immunoblotting either for 4 or 12 weeks treated mice (49).

Astellas Gene Therapies recently started a clinical trial phase 1/2 using this approach for the correction of DMD exon 2 duplication (NCT04240314) in three participants at Nationwide Children’s Hospital. The study aiming to access the systemic single dose delivery of scAAV9.U7.ACCA *via* peripheral vein injection is to be completed by November 2025. The scAAV9.U7.ACCA will cause the skipping of exon 2 during the splicing event to form a mRNA containing a single exon 2 or no exon 2 at all. Transcriptomic analyses indicated that scAAV9.U7.ACCA is not associated with important off target events in non-human primates (50) and in mice (51, 52). This approach is very encouraging for because of its potential safety in human. Although the preliminary clinical trial results are not yet available, the preclinical results described above seem to present this approach to be much promising that the ASOs approach where, whatever it has been granted accelerated approval, the highest editing percentage (6.55%) was shown with SRP-5051 (39).

### Mechanism Based on Nuclease Activity of CRISPR Associated Protein 9 Protein

The Clustered Regularly Interspaced Short Palindromic Repeats (CRISPR) technology is generally considered as new, simple, and efficient approach to cut and modify a DNA strand. The CRISPR associated protein 9 (Cas9) nuclease is guided to a specific region of the DNA through a sequence of 20 nucleotides called a single guide RNA (sgRNA) (53–55). This technology was first discovered in bacteria. Many bacteria and most archaea defend themselves from invading genomic elements from bacteriophage infections and plasmid transfer using this sophisticated adaptive immune system. After being exposed, a small fragment of foreign DNA is integrated into the CRISPR array in the host genome as a new spacer sequence, which provides a memory record of this infection. In future attacks by the same invaders, the foreign DNA fragment is recognized by the CRISPR RNAs and cleaved through enzymatic processing commonly known as endonucleolytic cleavage (53–55). There are six types of nucleases divided into two major classes.

Class 1 nucleases are composed of type I, III, and IV nucleases and require a large complex of several effector proteins for the RNA guided target cleavage system. Class 2 nucleases are composed of type II, V, and VI nucleases and require only one RNA guided endonuclease to mediate the cleavage. CRISPR/Cas9 are type II endonucleases and the most commonly used types of Cas proteins for genome editing. The most commonly used Cas9 proteins for gene editing are the *Streptococcus pyogenes* Cas9 (SpCas9) and *Staphylococcus aureus* Cas9 (SaCas9). SaCas9 is about 1 kb smaller than SpCas9 and can be easily packaged with its sgRNA into a single AAV vector in what has been called an all-in-one vector. To guide the Cas9 onto a specific genomic locus, a sgRNA containing a spacer sequence of 17–24 nucleotides is required. This sgRNA also contains a constant sequence, which forms a stable complex with the Cas9 (53–55). When performing gene editing, the Cas9 nuclease that is used must be chosen based on the nucleotide sequence surrounding the target site. Each Cas9 recognizes its own sequence of two to six nucleotides called the proto spacer adjacent motif (PAM) to bind to the DNA and subsequently causes an efficient DNA break.

Various technologies were derived from the original CRISPR/Cas9 system (CRISPR-induced double strand breaks (54, 55), Cytidine Base Editing (CBE) (56), Adenine Base Editing (ABE) (56), and PRIME Editing (PE) (57). Current efforts with these technologies are ongoing to investigate the feasibility of genetically modifying the *DMD* gene. These approaches seek to introduce permanent modifications in the genomes of DMD patients to efficiently restore the dystrophin expression. These modifications include the correction of nonsense mutations, the insertion of one or multiple nucleotides to reframe a frameshift mutation, the modification of splicing sites to favor exon skipping and reframing, etc. (58).

#### Clustered Regularly Interspaced Short Palindromic Repeats-Induced Double Strand Break

**FIGURE 3:**
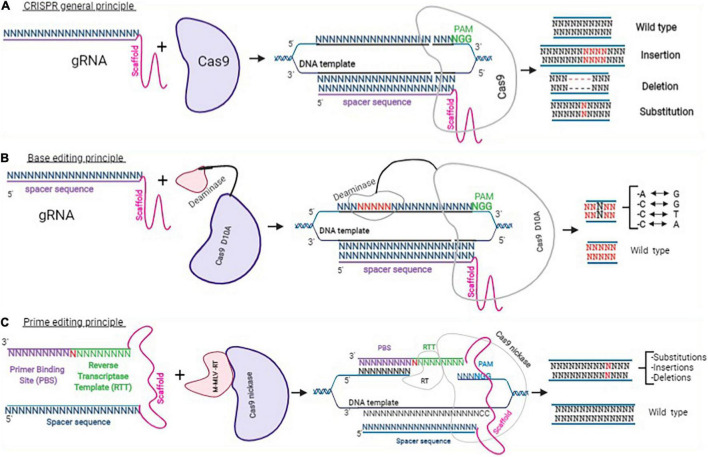
Clustered Regularly Interspaced Short Palindromic Repeats approaches. This figure adapted from Happi Mbakam et al. (58) represents the principle of CRISPR-Cas9 gene modification, which uses a guide RNA and a Cas9 to mediate the nuclease activity resulting in insertions, deletions, or INDELs **(A)**. Panel **(B)** represents the general mechanism for base editing, which uses a sgRNA (spacer sequence + scaffold) and a Cas9 nickase fused with a cytosine or an adenine deaminase. Depending on the deaminase used, after binding with a specific DNA template, a cytosine is chemically modified into a thymine or an adenosine into a guanine. Panel **(C)** shows a PE2 protein (a Cas9 nickase fused with an engineered reverse transcriptase enzyme from M-MLV) and a pegRNA. The pegRNA is an extended sgRNA including the spacer sequence and the constant sequence (scaffold), a reverse transcriptase template (RTT), and a primer binding site (PBS). The binding of PE2 and pegRNA with a DNA target permits to induce insertion, deletion, or substitution of a few nucleotides.

Depending on the genome modification that is required, 1 or 2 sgRNAs are required with a Cas9 nuclease to induce 1 or 2 double stranded breaks at 1 or 2 specific DNA sites (Figure 3A).

##### Exon Deletion

This approach was used by our research group to target sequences in exons 50 and 54 and in exons 47 and 58 to induce deletions of part of the DMD gene using 2 sgRNAs (59, 60). This generated a hybrid exon that not only restored the normal reading frame of the *DMD* mRNA, but also produced a dystrophin protein with a normal spectrin-like repeat structure. This was performed *in vitro* in DMD patient myoblasts carrying exon deletions and *in vivo* in the hDMDΔ52 mouse model (59, 60). About 50% of dystrophin expression was restored in the mdx23 mice by targeting the flanking regions of exons 21 and 23 to induce an in-frame deletion using 2 sgRNAs (61).

##### Exon Reframing

Koo et al. (62) successfully used this approach to mediate exon reframing using 1 sgRNA. Authors showed that 8 weeks post injection of 5 × 10^11^ vg/kg of AAV2/9 driving the expression of CjCas9 and one sgRNA in mdx23 mouse model of DMD resulted in 39 ± 4% dystrophin positive fibers in mice with one base pair insertion (cytidine nucleotide) and 28 ± 6% of mice carrying 14 base pairs deletion. The mice showed increase in muscle-specific maximal force. Min at al. (63) also showed that the insertion of a single adenine nucleotide at 5′ boundary of exon 45 lets to the reframing of that exon and the dystrophin expression.

##### Exon Skipping

This approach has also been experimented *in vitro* for the skipping of exon 23 in *DMD^mdx^* induced multipotent progenitor cells (iMPCs). Two sgRNAs were designed to target the splice donor site of exon 23 mediating the skipping of exon 23 during the splice event. The corrected cells restored the dystrophin expression *in vivo* and contributed to the muscle stem cell reservoir in *DMD^mdx^* (64). Min et al. (63) used 1sgRNA to target the 5′ junction of exon 45 in a mouse carrying the deletion of exon 44 to mediate the skipping of exon 45. By skipping the exon 45, the strategy permitted to link the exon 43 to exon 46 to restore the reading frame. A systemic delivery of Cas9 and sgRNAs through peritoneal injection using two AAV9 vectors (5 × 10^13^ vg/kg at a ratio of 1:1) resulted in 94, 90, and 95% dystrophin expression observed by immunostaining respectively in TA, triceps, and diaphragm muscles followed by improvement in mouse muscle function. A ratio of 1 (Cas9):10 (sgRNA) was used to achieve similar results in cardiomyocytes.

However, this CRISPR double strand break technique promotes frequent non-specific insertions and deletions following off-target DNA cleavages. In the Min et al. study, up to 0.2 and 1.1% inverted terminal repeat integrations were reported respectively at the genomic and the mRNA levels. Koo et al. also reported up to 8 ± 0.7% INDELs. Happi Mbakam et al. recently presented an update of the CRISPR-Cas9 gene therapy for dystrophin replacement in DMD (58).

#### Base Editing

This approach uses an adenine or cytidine deaminase coupled to a Cas9 nickase (a modified Cas9, which can only cut one DNA strand) to deaminate a cytidine (C to T modification) or an adenine (A to G modification) after being guided to that specific genomic region by a sgRNA (Figure 3B) (65). This system has been used in the adult *mdx4cv* mouse, a dystrophic mouse model of DMD carrying a premature stop codon in exon 53 of the *DMD* gene, to demonstrate that base editing can restore dystrophin expression. The authors of that experiment showed that the systemic delivery of the adenine base editor (ABE) using an improved AAV vector (AAV-iNG) led to long-term and efficient restoration of up to 80% dystrophin and functional improvement of dystrophic heart (66). However, this technique is not precise enough because, other adenines or cytidines present in the editing window are susceptible to be also deaminated (65). CBEs and ABEs are also recognized to generate other off-target mutations (67).

#### Prime Editing

Prime editing is one of the most recent, efficient, and precise genome editing technology to be developed so far. It uses a prime editor (PE) and a prime editing guide RNA (pegRNA) (Figure 3C). The prime editor is made of a Cas9 nickase protein fused through a flexible linker to an engineered Murine Leukemia Virus Reverse transcriptase (RT). The pegRNA is a modified sgRNA, it contains the spacer sequence of the sgRNA (a sequence of 20 nucleotides designed to target a specific DNA locus), the scaffold of the sgRNA (which permits it to bind to the Cas9 protein). However, the pegRNA also contains a 3′ extension made of a primer binding site (PBS) and a reverse transcriptase template (RTT). The PBS sequence is designed to serve as primer to start the synthesis of a complementary DNA strand by the RT. The RTT sequence serves as a template for the synthesis by the RT of the intended complementary strand containing the desired modification(s) (57). The pegRNA can, in principle, change any nucleotide for any other nucleotide with precision. This technique can therefore correct point mutation, and either eliminate or insert a few nucleotides to permanently modify the genome of target cells (57, 66, 68–71). Many ongoing experiments are demonstrating the capacity of this approach to permanently correct the DMD gene as well as other genetic mutations responsible for many other hereditary diseases (69, 72).

### Mechanism Based on Progenitor Modulation

Also considered as a stem cell disease, the DMD disease progression and severity is strongly associate to the capacity of myogenic progenitors to maintain the regeneration, growth and repair of skeletal muscle (20). Satellite cells, the prototype of muscle stem cells, have been identified as the primary quiescent progenitor for myofibers (73). The cell environment has been identified to play a key role in the maintenance of the satellite cell niche and the performance of these stem cells (74). Many other progenitors such as mesoangioblasts (75), pericytes (76), side population cells (77), muscle derived stem cells (78), PW1+ interstitial cells (79), and fibro-adipogenic progenitors (150) have also been investigated for muscle regeneration.

Histone deacetylase inhibitors (HDACi), such as valproic acid, phenylbutyrate, givinostat (ITF2357), and suberoylanilide hydroxamic acid, have been tested in DMD mouse models. Although, promising results have been obtained in mdx mice (80), the safety and tolerability of these drugs in the pediatric population remains the main concern for their use in clinical trials. However, the pharmacological effects of Givinostat, a class I and II HDACi, in pediatric patients are already known (81). By blocking the histone deacetylase enzyme, Givinostat permitted the activation of the follistatin gene leading to muscle mass increase. That effect also prevented muscle degeneration, reducing the adipogenesis process and fibrotic tissue accumulation.

Interim results from phase 1 and 2 clinical trials (NCT01761292) sponsored by Italfarmaco Inc. involving 20 DMD patients aged 7–10 years showed that Givinostat induced a significant reduction of the amount of fibrotic tissues, tissue necrosis and fatty replacement. It also significantly increased the fraction of muscle tissue in the biopsies. No improvement was observed in functional tests (82). The NCT01761292 trial has been extended to phase 2/3 clinical trial (NCT03373968) for the study of long-term safety and tolerability of Givinostat. The study is expected to be ended by December 2023. Italfarmaco Inc. communicated in February 2021 that boys from this study continued to show delayed disease progression (83). The ongoing phase 3 pivotal trial (NCT02851797) has enrolled 179 patients aged 6–17 years. The aim of this trial is to evaluate the efficacy and the safety of Givinostat and the preliminary results is expected in second quarter of 2022 (83). Givinostat has been approved by the European Medicine Agency for the treatment of DMD (84). FDA also granted in October 2020 a Rare Pediatric Disease designation to Givinostat for the treatment of DMD (83).

### Gene Replacement

The gene replacement approach consists of transferring a functional copy of complete or truncated dystrophin gene into myofibers to restore the muscle strength in DMD patients. The *DMD* gene is one of the biggest genes measuring about 2.2 million base pairs (85). This large size makes it difficult to package. In this approach, the full-length dystrophin cDNA and mini or micro-dystrophin constructs, are investigated to develop treatments for DMD.

#### Artificial Chromosome Transfer of the Dystrophin Gene

Human artificial chromosome (HAC) is a kind of multipotent vector derived from native or synthetic chromosome, able to deliver a full length *DMD* gene in mice and eventually in humans (151). Benedetti et al. (86) developed a novel generation of HAC (DYS-HAC 2) eliminating potential immunogenic products such as the enhanced green fluorescent protein (EGFP), blasticidin (Bsd), Herpes Simplex Virus type 1-thymidine kinase (HSV1-TK), and hypoxanthine-guanine phosphoribosyl-transferase (HPRT) from DYS-HAC 1 (86). DYS-HAC 2 permits genomic integration, reversible immortalization, genetic correction, additional dystrophin expression, inducible differentiation, and cell death control.

This approach presents at least two important advantages for DMD gene therapy. The first is the ability to carry large DNA sequences that could lead to physiological expression of genes with complex transcriptional regulation such as the *DMD* gene. The second advantage is the episomal maintenance of a single gene copy, preventing the risk of oncogenic insertion (86). However, some limitations (low formation efficiency, the structural complexity, tissue target efficiency, and a stability problem) explain why this approach did not yet move forward to clinical trial.

#### Full Length Dystrophin

Meng et al. showed that DMD myoblasts transduced with a foamy virus vector expressing the full-length dystrophin transplanted intramuscularly into *mdx* nude mice, participated in muscle regeneration leading to the expression of full-length and functional dystrophin proteins (87).

#### Micro-Dystrophin

**FIGURE 4:**
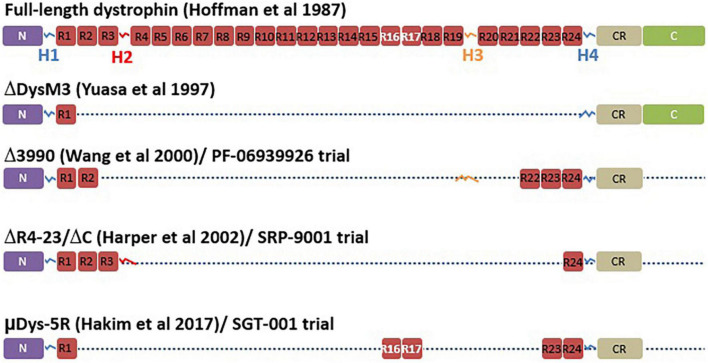
Micro-dystrophin variants. This figure adapted from Duan et al. (147) represents the full-length dystrophin with different domains including the N-terminal domain (N), the four hinges (H), the central rod domain (R), the cysteine rich domain (CR), and the C-terminal domain (C). The figure also illustrates the different versions of micro-dystrophin with lacking domains in use in different clinical trials including the initial micro-dystrophin constructed by Yuasa et al. (149), the Δ3990 used for the development of PF-06939926, the (ΔDysM3), the ΔR4-23/ΔC used for the development of SRP-9001, and finally the μDys-5R used for the development of SGT-001.

Mini or micro-dystrophin are short versions of the *DMD* gene made of at least N terminal domain and different parts of the *DMD* sequences between these N and C terminal domains (Figure 4). Several micro-dystrophins and different AAVs serotypes have been developed through decades of research on this approach (88). Nowadays, three micro-dystrophins are used in clinical trials: the five-repeat micro-dystrophin D3990 (89) (Pfizer Inc. trial for PF-06939926), the four-repeat micro-dystrophin ΔR4–23/ΔC (90) (Sarepta Inc. trial for SRP-9001) and the five-repeat micro-dystrophin mDys5R (91) (Solid GT Inc. trial for SGT-001). All the three transgenes have in common at least the N-terminal domain, the cysteine-rich domain, the spectrin-like repeats 1 and 24 (R1 and R24) and Hinges 1 and 4 (H1 and H4). All of them lack the C-terminal domain spanning from exons 71 to 79. The C-terminal domain contain the alpha1 and beta1 syntrophin biding sites and the dystrobrevin biding site, which play important role in signalization pathways and the maintenance of dystrophin complex (3). This domain has been identified to have a protective role in heart (92). Wang et al. (93) showed that compared to full-length dystrophin, micro-dystrophin (ΔR4–23/ΔC) has altered association with alpha and beta syntrophins and cavins in *mdx^5cv^* mouse hearth. The authors also indicated that the expression of micro-dystrophin in *mdx^5cv^* mice prevented the development of cardiac histopathology but did not rescue membrane localization of cavins nor did it normalize ERK signaling (93). Bourdon et al. (94) recently showed in DMD*^mdx^* rats that the ΔR4-23/ΔCT expression is sufficient to restore the interactions of most dystrophin associated protein complex (DAPC) partners in skeletal and cardiac muscles. In contrast, the addition of C terminal domain significantly increases the restoration of that interaction. The micro-dystrophin D3990 lack the nNOS binding domain and H2. The micro-dystrophin DR4–23/DC lack the nNOS binding domain and H3. The micro-dystrophin mDys5R lack H2 and H3. The H2 influence functional capacity of micro-dystrophin in mouse model (3). The nNOS is involved in important signaling transduction pathways and produces NO leading to vasodilatation which help to provide oxygen and nutrients to muscle cells (3). It is evident that each domain removed from the native dystrophin gene to build micro-dystrophin plays a role in the dynamism of the DAPC. To which extend the removal of the so qualified “unnecessary domains” in micro-dystrophin impacts the membrane dynamism and is not clear cut.

In 2020, Mendell et al. (95) published a preliminary result from the phase 1/2 clinical trial study (NCT03375164) sponsored by Sarepta Therapeutics Inc. that aimed to evaluate the safety and tolerability of intravenous SRP-9001 in DMD patients. This treatment involved the use of a recombinant adeno-associated virus (AAV) serotype rh74 (rAAVrh74) and codon-optimized human micro-dystrophin sequence expressed using a skeletal and cardiac muscle-specific promoter with enhanced cardiac expression (MHCK7). The micro-dystrophin sequence was made of N-terminus actin binding domain, spectrin-like repeats 1 to 3 and 24, hinges 1, 2, and 4, the cysteine-rich domain and the C-terminus domain. Four boys aged 4–7 years with exon deletions (46–50, 46–49, partial deletion exon 44) or a premature stop codon in exon 27 were enrolled. Patients received single intravenous infusion of 2 × 10^14^ vg/kg rAAVrh74.MHCK7 micro-dystrophin. The immunochemistry of muscle biopsy showed 81.2% of muscle fibers expressing micro-dystrophin with an intensity of 96% at the sarcolemma. Functional improvement was also observed in North Star Ambulatory Assessment (NSAA) scores. The patients were followed until March 2021 with the results yet to be published. A parallel randomized, double blind, placebo-controlled study of SRP-9001 (NCT03769116) involving 41 participants is planned to be completed by April 2026. A minimal fat infiltration was further confirmed in a case control study (96) assessing the association between treatment with rAAVrh74 and muscle quality in three patients previously involved in the Mendell et al. study (95).

There is another ongoing randomized, placebo-controlled phase 3 clinical trial sponsored by Pfizer Inc., which seeks to evaluate the efficacy and safety of PF-06939926 (NCT04281485) in 99 boys aged 4–7 years old. The mini-dystrophin gene is delivered by an AAV9 serotype, which has good affinity to skeletal muscle, nervous, heart, liver, and lung tissues (97). The study is expected to be completed by September 2028. However, in December 2021, FDA imposed a clinical hold after the death of one patient participating the non-ambulatory cohort of the phase 1b trial (NCT03362502) (98). The trial was a multicenter, open-label, single ascending dose study to evaluate the safety and tolerability of PF-06939926 in ambulatory and non-ambulatory subjects with DMD. The preliminary results presented by Pfizer Inc. in 2019 were promising and some observed adverse effects were resolved in approximately 2 weeks (99).

In September 2021, Solid Bioscience LLC reported 1.5-year data of their IGNITE trial phase 1/2 (NCT03368742), which was a controlled, open-label, single-ascending dose study to evaluate the safety, the tolerability and efficacy of SGT-001 in adolescents and children with DMD. The company indicated that SGT-001, which is a rAAV9 encoded micro-dystrophin, showed functional benefits through NSAA, 6MWT, and Forced Vital Capacity (FVC) compared to natural history data after 1.5-post treatment (100).

Some recent trials in this approach have been summarized in Table 2. The results of Mendell trial (NCT03375164), showed a great promise of this gene transfer approach although the observed mild and moderate side effects (95) and a death in Pfizer trail (NCT03362502). Specific questions about the AAVs and transgene safety and the long-term assessment in this respect need to be addressed. There is no evidence about the long-term micro-dystrophin related truncated dystrophin expression in DMD patients.

**TABLE 2:** Summary of clinical trials of gene transfer for DMD.

AAV serotype	Study phase	Status	Period	Trial number	Result available	Number of patients	Study title
AAV9	1	Active, not recruiting	January 2018– July 2026	NCT03362502	No	30	A Study to Evaluate the Safety and Tolerability of PF-06939926 Gene Therapy in Duchenne Muscular Dystrophy
rAAVrh74	1 and 2	Active, not recruiting	November 2017– November 2021	NCT03333590	No	6	Gene Transfer Clinical Trial to Deliver rAAVrh74.MCK.GALGT2 for Duchenne Muscular Dystrophy
rAAVrh74	1 and 2	Active, not recruiting	January 2018– April 2023	NCT03375164	No	4	Systemic Gene Delivery Clinical Trial for Duchenne Muscular Dystrophy (DMD)
rAAVrh74	1	Completed	March 2015– September 2017	NCT02376816	Yes	2	Clinical Intramuscular Gene Transfer Trial of rAAVrh74.MCK.Micro-Dystrophin to Patients with Duchenne Muscular Dystrophy
rAAVrh74	2	Active, not recruiting	December 2018– April 2026	NCT03769116	No	41	A Randomized, Double-blind, Placebo-controlled Study of SRP-9001 for Duchenne Muscular Dystrophy (DMD)
rAAVrh74	1	Recruiting	November 2020– March 2026	NCT04626674	No	20	A Gene Delivery Study to Evaluate the Safety of and Expression From SRP-9001 in Duchenne Muscular Dystrophy (DMD)
rAAV9	1 and 2	Recruiting	December 2017– April 2027	NCT03368742	No	16	Micro-dystrophin Gene Transfer Study in Adolescents and Children With DMD
rAAV9	3	Recruiting	November 2020– October 2027	NCT04281485	No	99	A Phase 3 Study to Evaluate the Safety and Efficacy of PF-06939926 for the Treatment of Duchenne Muscular Dystrophy
scAAV9	1 and 2	Enrolling by invitation	January 2020– January 2025	NCT04240314	No	3	AAV9 U7snRNA Gene Therapy to Treat Boys with DMD Exon 2 Duplications

### Mechanism Based on Skeletal Muscle Cell Transplantation

This approach is investigated for DMD and other myopathies. It is based on a specific healthy donor cell transplantation to a specific DMD patient to repair the damaged organ or to repopulate the satellite cell pool and enable the dystrophin expression (101). The engraftment of human cells in an immunodeficient mouse model requires preliminary mechanical, irradiation or chemically induced muscle damage to create a niche (102). The transplanted cells have to be able to proliferate, differentiate and integrate into the host muscle (103). The cell types usually transplanted are satellite cells, induced pluripotent stem cells, mesoangioblasts, pericytes, myoendothelial cells, and myoblasts derived from satellites cells (101). Cells can be delivered by multiple intra-muscular injections, repeated cell delivery or systemic delivery (102). In a preclinical study involving seven non-human primates, Skuk and Tremblay showed that the intra-arterial (femoral arteries) injection of myoblasts contribute to myofiber regeneration but only in muscle sites that were damaged at the time of cell injection (104). There is no recent update about the human myoblast transplantation to restore the dystrophin. Some reasons could be pointing out. The proliferation of myoblasts is labor intensive. The myoblasts injected in a muscle remain at the injection sites and thus because of large tissue surface hundreds of injection sits are required making this treatment difficult for the patients (105). Moreover, r transplanted myoblasts and finally, a high percentage of myoblasts usually died at the injection sites (106). A study of the kinetic of death of transplanted myoblasts in non-human primates over three weeks noted that transplanted myoblasts died at a significantly higher rate during the first week due to an inflammatory response that remains a critical challenge to be resolved for intra-muscular skeletal muscle cell transplantation (107).

A clinical trial conducted with nine immunosuppressed DMD patients to test a protocol including a high density of injection trajectories in skeletal muscles indicated that the transplanted cultured myoblasts from one of the patient’s parent permitted the expression of the donor dystrophin in 3.5 and up to 26% of the muscle fibers in eight patients (108). Thus, this clinical trial conducted in immunosuppressed DMD patients showed evidence that allo-transplantation under Tacrolimus immunosuppression of normal myoblasts permitted their fusion with patient myofibers leading to dystrophin expression. However, the presence of muscle fibers expressing the donor dystrophin was observed only close to the injection sites. Other investigational studies by the same authors demonstrated the pertinence and the relevance of this approach following different methodologies, which resulted in 34.5% dystrophin positive fibers in one DMD patient (105, 109).

A single blinded trial conducted in various hospitals in India with 11 participants showed the proof of principle that the transplantation of human umbilical cord mesenchymal stem cells stabilized the muscle power with no serious adverse effect (110).

### Utrophin Upregulation

Utrophin is known as dystrophin homolog mostly found at the neuromuscular junction. It possess similarities with dystrophin in sequences and protein binding properties (111). Utrophin A is the predominant isoform and is found in the myotendinous junction of adult muscles and at the sarcolemma of regenerating myofibers (111). It has been shown that the activation of *UTRN-A* promoter mediates the upregulation of *UTRN* expression in mdx mice (112, 113). Some post-transcriptional effectors of utrophin upregulation have also been identified in a mouse model of DMD using an utrophin 5′/3′UTR reporter assay (114). Sengupta et al. (115) used the CRISPR-Cas9 technic to delete 500 bp IMTR containing five miRNAs binding sites within the UTRN in DMD patients derived human pluripotent stem cells (hiPSCs). The deletion of that sequence resulted in a twofold increase in utrophin level, which permitted to improve the dystrophin glycoprotein complex restoration. Some authors also successfully used this approach (112, 113, 116). The upregulation of utrophin as therapeutic approach helps in the maintenance of muscle stability and integrity thus, might functionally compensates the lack of dystrophin in DMD. Although the upregulation of utrophin has benefit in animal DMD models, there is no evidence about the impact of the persistence of that over-expression. However, this approach may be beneficial for all DMD cases since it is not mutation type-related and the UTRN is present in all DMD patients.

Clinical trials supported by Summit Therapeutics Inc. including the phase 1 placebo-controlled randomized trial (NCT02383511) in 12 healthy male volunteers, and the phase 1b placebo-controlled, randomized, double-blind study (NCT02056808) in 12 boys with DMD, demonstrated that Ezutromid (SMT C1100) was safe and well-tolerated by participants (117). The phase 2 trial (NCT02858362) involving 43 participants and consider as proof of concept study to assess activity and safety of SMT C1100 in boys with DMD was ended because of lack of efficacy (118).

## Side Effects of Drugs

Each drug development approach for DMD has the main objective to develop a suitable molecule with long-term effectiveness and safety for DMD patients. At least two major reasons including the incapacity of the drug to mediate dystrophin expression and the harmful side effects of the drugs could explain the fact that some potential drug developments for DMD did not move forward. Gentamicin is one example of read through molecule that seemed to mediate interesting level of dystrophin expression but associated with important side effects such as ototoxicity, neurotoxicity, nephrotoxicity, harmful injection site reactions, nausea, stomach upset, vomiting, etc. (18, 24). ASO therapeutics mediating exon skipping also highlighted several toxicities including upper respiratory tract infections, cough, fever, headache, joint pain, pain in the mouth and throat, blood disorders, hyperglycemia, complement and coagulation cascades activation, nephrotoxicity, hypotension, etc., attributed to the chemical structure of these molecules (119). With regards to the level of dystrophin restoration that ranged from 0.2 to 6.5% editing percentages versus the side effects observed, it is questionable how easy most of these ASO therapeutics have been granted accelerated approvals for the treatment of DMD. PRO044 is an example of molecules that showed some of these side effects in DMD patients (NCT01037309). Most of times, drug developments for DMD are paused because of ineffectiveness of dystrophin expression.

The dystrophin replacement is usually associated with immune response both against dystrophin (120, 121) and AAV vector (122, 123) used to deliver the micro-gene. Mendell et al. (124) raised attention a decade ago about the consideration of cellular immunity against self and non-self dystrophin epitopes for the mini dystrophin gene transfer. As well, a 24 months study carried out in GRMD dogs for the dystrophin restauration through mini-dystrophin cMD1 gene transfer using the rAAVs2/8 indicated a transient humoral immune response against cMD1 1–2 months post injection. These reactions become undetectable after 8 months except for one dog who showed a persistent IgG response to cMD1 (121). These authors also indicated a persistent immune response against the AAV8 capsid (121). The use of immunomodulatory drugs such as rituximab and VBP6 might completely abrogate the cellular immune response against micro-dystrophin and partially the interferon gamma response (120).

The *in vivo* AAVs related gene transfer has been shown to be linked to adverse events including neurotoxicity, hepatotoxicity, myocarditis, oncogenicity, and thrombotic microangiopathy (125). Third death were recorded in 2020 because of hepatotoxicity during the ASPIRO clinical trial (NCT03199469) phase 1/2 for the treatment of X-Linked Myotubular Myopathy (126). The patients were treated with high dose of 3.5 × 10^14^ vg/kg of the AT132 which is an AAV8 vector containing a functional copy of the human MTM1 (hMTM1) gene. Despite the predicted lower dose of 1.3 × 10^14^ vg/kg that supposed to be safe, four other deaths were recorded in September 2021 (127). The chromosomal integration also represent a non-negligeable risk for the use of some AAV serotypes (128). This evidence highlights serious concerns about the prior validation of AAVs for the prevention of patient health.

## Delivery Methods

The choice of drug delivery systems is a challenging question in drug development experiments. Although they allow for good precision and efficiency *in vitro*, molecules such as programable nucleases or nucleic acid are always challenging to deliver to a target site *in vivo*. These difficulties account for the low number of clinical studies. The choice of one method over another always depends on characteristics such as immunogenicity, packaging capacity, integration ability, long or short-term expression ability, etc. Nowadays, many approaches have been investigated such as: (1) biological delivery approaches including viral delivery systems (129) and extracellular vesicle (EV) delivery systems (130), (2) chemical delivery approaches including nanoparticles, peptide-based, and lipid particles (131), (3) physical delivery approaches including injections, electroporation, ultrasound, ballistic DNA, photoporation, magnetofection, mechanical massage, and hydrostatic pressure (131). Here we will only discuss the biological approaches explored by researchers to deliver large molecules to human or animal models.

### Viral Delivery System

Many viral delivery systems are used for gene therapy including AAV, adenovirus (AdV), lentivirus, and bacterial phages (129). AAVs are frequently used in gene therapy experiments, and thus they are currently the most well characterized virus for human therapy. Their serotype diversity allows them to preferentially target a specific organ or biological system (132). Their limited size capacity, which was an important issue for gene therapy, was recently solved by the development of trans-splicing dual or triple AAV vectors but with reduced expression efficiency (133, 134). Dual AAVs coding for proteins with intein sequences have also been used because these inteins can fuse to each other and exclude themselves in the process of making the fusion protein (135).

### Extracellular Vesicle Delivery System

Extracellular vesicles represent a new alternative for the delivery of nucleic acids and proteins. EVs are small lipid bilayer particles that are released by cells. They play a critical role in cell-to-cell communication by carrying and transferring their contents to other cells (130). Their contents and function strictly depend on their biogenesis. EVs can be divided into many sub-populations: micro-vesicles, exosomes, apoptotic vesicles, ectosomes, and lysosomes (130). They can be used as biomarkers of diseases by observing their contents and extrapolating the states of the cells producing them from there (130). The EV delivery method is gaining interest in science because of their natural potential to carry macromolecules such as Cas9 components. Bobis-Wozowicz et al. showed the proof of concept that programable nucleases can be packaged into EVs for genomic target editing (136). Gee et al. also demonstrated that EVs can be used as a delivery method for the CRISPR/Cas9 protein and sgRNA for exon 45 skipping in DMD induced pluripotent stem cells with 90% efficiency (137).

## Perspectives

The therapeutic approach consisting of read through the mutation initially seemed to be the most effective drug development approach to handle DMD nonsense mutations allowing significant phenotypic improvement. However, success rate in this approach remains very low and usually combined adverse effects observed in treated patients. These adverse effects prevalently include ototoxicity, neurotoxicity, nephrotoxicity, harmful injection site reactions, nausea, stomach upset, etc., depending on the molecule or the strategy (18, 27). Another approach allowing exon skipping using ASOs has achieved up to 5% dystrophin expression after 24 weeks of treatment (36), which has been considerably improved to 6.5% mean dystrophin production for only three doses of a monthly administration of 30 mg/kg SRP-5051 in DMD patients with amenable exon 51 skipping in the phase 2 momentum trial of SRP-5051 (39). Many chemical improvements have been made to this drug approach to reduce adverse effects minimizing the toxicity level for improved tolerability (95). Improvements also made the drug more stable increasing the half-life to enlarge the time between two uptakes. They also permit to increase the drug affinity and specificity to the target as well as the level of dystrophin production (39, 138). One of the most recent and more promising modification made by Sarepta therapeutics for antisense therapeutics is the addition of polypeptides to PMO to form the next generation PPMO (39). Gene replacement approaches are also interesting since they would permit long-term dystrophin expression. The most advanced gene replacement approach inducing a robust dystrophin expression consists of transferring a functional copy of a micro-dystrophin gene with a recombinant AAV serotype rh74 (rAAVrh74) and codon-optimized human micro-dystrophin sequence expressed using a skeletal and cardiac muscle-specific promoter with enhanced cardiac expression (MHCK7) (95). There are some ongoing phase 3 clinical trials for this approach (NCT03769116 and NCT03362502), but results are not yet available. However, a previous trial reported some cases of advert effects mostly included nausea, vomiting and an elevated transaminase levels that should be taken into account for subsequent study designs (95).

One of the big challenges in the gene replacement approach is the problem of transgene persistence and sustainability of the treatment. The *in vivo* AAVs persist in episomal forms. The therapeutic efficacy can then be significantly reduced or lost when cells proliferate (139). Since the persistence depend on the division rate, the transgene expression persistence depends on the tissue type. Nakai et al. (140) showed that up to 92% of rAAV injected in mice portal vein were loss after hepatectomy at 6–12 weeks post injection. Authors suggested that episomal rAAVs represent the major form of gene expression in the liver. Efforts are put together to develop vectors that can be less genotoxic and more persistent in episomal forms (141, 142). Such effort might be beneficial for diseases requiring long-term robust expression of transgene. The DMD patients mostly die by the age of 30 because of cardiorespiratory complications (13, 14). The DMD patients are often diagnosed late by their teen age. The capacity of micro-dystrophin to prevent such cardiorespiratory complications is currently unknown. The existing animal models for DMD are not appropriate for such a study because they die before the disease progression into cardiopathy. To address this gap, Howard et al. (143) recently developed a dystrophic dko mouse model with transgenic correction of skeletal muscles that progresses into late-stage heart failure over 12 months in a series of steps similar to human DMD cardiomyopathy. Authors showed for the first time that micro-dystrophin effectively prevents cardiac failure in mouse.

The gene modification approach using CRISPR/Cas9 technology is evolving rapidly and gaining interest for different possibilities to modify the human genome. However, it leads to frequent micro-insertions or micro-deletions (INDELs) (59). Modifications of the Cas9 gene resulted in the development of Cas9 variants with PAMless requirement, nickase and/or high-fidelity activity (144). Nowadays, there are new nuclease variants coupled with cytidine or adenine deaminase to change cytidine into thymine and adenine into guanine, respectively. This permitted the specific modification of DMD nonsense mutations with editing efficiency of 80% in the mdx^4*cv*^ mice. Off-target mutations, however, are also associated with this technology, both in the conversion window and in the transcriptome of the cells. The most recent and promising gene editing technology is the CRISPR/Cas9 Prime editing technique that can change any nucleotide into any other nucleotide, as well as insert or delete nucleotides in a specific sequence (57). That technology has recently been modified for more efficiency (69, 70, 145). There are some examples, which have served as proofs of principle, that Prime editing can be used to introduce specific point mutations in many exons of the *DMD* gene (72). Translation to human trials for CRISPR/Cas9 derived technologies is very difficult to implement because of a set of challenges including pre-existing immune responses to Cas9, the effects of repetitive expositions, the genome stability and off target effects of the protein, the optimization for the transient expression of Cas9, etc.

Adeno-associated viruses have long been used as viral delivery method for gene therapy approaches. However, AAVs induce immune responses and thus, patients undergoing treatment using AAVs will not be able to participate in other trials using the same vector (129). This serious issue might be handled by the removal of AAVs neutralizing antibodies by plasmaphereses in AAV seropositive candidates prior to treatment (122) and by immunomodulation of anti-AAV precursor (120, 123). Another approach to solve this anti-AAV issue is the development of AAV serotypes that are less immunogenic and cannot be recognized by the host immune system (146). AAVs also have limited packaging size, a drawback which is partially circumvented using trans-splicing technics with dual or triple AAV systems to package large DNA sequences. However, the trans-splicing methods are not as efficient as the use of single AAVs (133, 134). EVs represent a potential alternative for gene therapy cargo delivery. EVs are naturally produced by the human body and can be used to transfer large molecules (136).

## Conclusion

**FIGURE 5:**
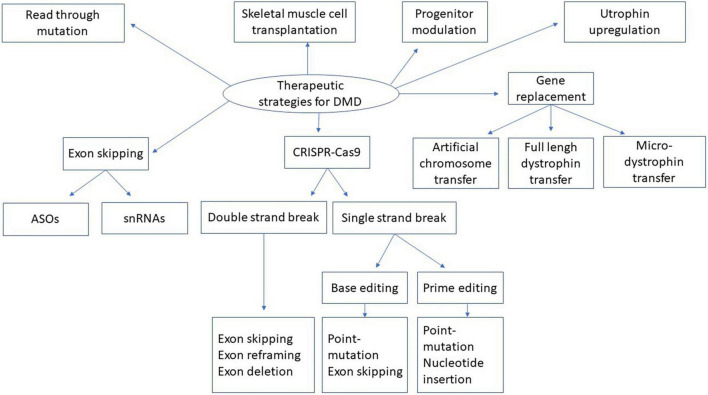
Therapeutic strategies for DMD. This figure represents the summary of different strategies described in the present manuscript for dystrophin replacement in DMD. snRNA means small nuclear ribonucleic acid. ASOs means antisense oligonucleotides. CRISPR-Cas9 means Clustered Regularly Interspaced Short Palindromic Repeats, CRISPR-associated protein 9.

Duchenne muscular dystrophy, as a hereditary rare disease, causes serious burdens, and suffering for both patients and their families. The increased knowledge about the disease and its etiology has led to a better understanding and development of pharmacological and clinical strategies to help patients. Nowadays, interesting therapeutic approaches (Figure 5) and combined strategies for phenotypic improvement, transitive, or long-term dystrophin expression under the sarcolemma are increasing and giving hope to those afflicted with this disease. Some of these DMD treatments have already obtained pre-authorization to treat some DMD patients. Improvements are still required to increase the effectiveness and long-term safety of these treatments.

## Author Contributions

CH designed and wrote the manuscript. GL revised and improved the English spelling. JT revised and supervised the manuscript. All authors contributed to the article and approved the submitted version.

## Conflict of Interest

The authors declare that the research was conducted in the absence of any commercial or financial relationships that could be construed as a potential conflict of interest.

## Publisher’s Note

All claims expressed in this article are solely those of the authors and do not necessarily represent those of their affiliated organizations, or those of the publisher, the editors and the reviewers. Any product that may be evaluated in this article, or claim that may be made by its manufacturer, is not guaranteed or endorsed by the publisher.

## References

[1] Blake DJ Weir A Newey SE Davies KE. Function and genetics of dystrophin and dystrophin-related proteins in muscle. *Physiol Rev.* (2002) 82:291–329. 10.1152/physrev.00028.2001 11917091

[2] Zhang Y Li H Min Y-L Sanchez-Ortiz E Huang J Mireault AA Enhanced CRISPR-Cas9 correction of duchenne muscular dystrophy in mice by a self-complementary AAV delivery system. *Sci Adv.* (2020) 6:eaay6812. 10.1126/sciadv.aay6812 32128412 PMC7030925

[3] Gao Q McNally EM. The dystrophin complex: structure, function and implications for therapy. *Compr Physiol.* (2015) 5:1223–39. 10.1002/cphy.c140048 26140716 PMC4767260

[4] Fealey ME Horn B Coffman C Miller R Lin AY Thompson AR Dynamics of dystrophin’s actin-binding domain. *Biophys J.* (2018) 115:445–54. 10.1016/j.bpj.2018.05.039 30007583 PMC6084637

[5] Mias-Lucquin D Dos Santos Morais R Chéron A Lagarrigue M Winder SJ Chenuel T How the central domain of dystrophin acts to bridge F-actin to sarcolemmal lipids. *J Struct Biol.* (2020) 209:107411. 10.1016/j.jsb.2019.107411 31689503

[6] Bies RD Caskey CT Fenwick R. An intact cysteine-rich domain is required for dystrophin function. *J Clin Invest.* (1992) 90:666–72. 10.1172/JCI115909 1644931 PMC443149

[7] Nowak KJ Davies KE. Duchenne muscular dystrophy and dystrophin: pathogenesis and opportunities for treatment. *EMBO Rep.* (2004) 5:872–6. 10.1038/sj.embor.7400221 15470384 PMC1299132

[8] Dystrophin isoforms. *Dystrophin Isoforms and Their Expression [Internet].* (2006). Available online at: https://www.dmd.nl/isoforms.html (accessed May 29, 2021).

[9] Crisafulli S Sultana J Fontana A Salvo F Messina S Trifirò G. Global epidemiology of duchenne muscular dystrophy: an updated systematic review and meta-analysis. *Orphanet J Rare Dis.* (2020) 15:141. 10.1186/s13023-020-01430-8 32503598 PMC7275323

[10] Ryder S Leadley RM Armstrong N Westwood M de Kock S Butt T The burden, epidemiology, costs and treatment for duchenne muscular dystrophy: an evidence review. *Orphanet J Rare Dis.* (2017) 12:79. 10.1186/s13023-017-0631-3 28446219 PMC5405509

[11] Bladen CL Salgado D Monges S Foncuberta ME Kekou K Kosma K The TREAT-NMD DMD global database: analysis of more than 7,000 duchenne muscular dystrophy mutations. *Hum Mutat.* (2015) 36:395–402. 10.1002/humu.22758 25604253 PMC4405042

[12] Liechti-Gallati S Koenig M Kunkel LM Frey D Boltshauser E Schneider V Molecular deletion patterns in duchenne and becker type muscular dystrophy. *Hum Genet.* (1989) 81:343–8. 10.1007/BF00283688 2784778

[13] Ciafaloni E Kumar A Liu K Pandya S Westfield C Fox DJ Age at onset of first signs or symptoms predicts age at loss of ambulation in duchenne and becker muscular dystrophy: data from the MD STARnet. *J Pediatr Rehabil Med.* (2016) 9:5–11. 10.3233/PRM-160361 26966795 PMC5868738

[14] NIH. *Duchenne Muscular Dystrophy | Genetic and Rare Diseases Information Center (GARD) – an NCATS Program [Internet].* (2020). Available online at: https://rarediseases.info.nih.gov/diseases/6291/duchenne-muscular-dystrophy (accessed February 25, 2021).

[15] Li H Xiao L Wang L Lin J Luo M Chen M HLA polymorphism affects risk of de novo mutation of dystrophin gene and clinical severity of duchenne muscular dystrophy in a Southern Chinese population. *Front Neurol.* (2018) 9:970. 10.3389/fneur.2018.00970 30498470 PMC6249334

[16] Nakamura A Yoshida K Fukushima K Ueda H Urasawa N Koyama J Follow-up of three patients with a large in-frame deletion of exons 45–55 in the duchenne muscular dystrophy (DMD) gene. *J Clin Neurosci.* (2008) 15:757–63. 10.1016/j.jocn.2006.12.012 18261911

[17] England SB Nicholson LVB Johnson MA Forrest SM Love DR Zubrzycka-Gaarn EE Very mild muscular dystrophy associated with the deletion of 46% of dystrophin. *Nature.* (1990) 343:180–2. 10.1038/343180a0 2404210

[18] Malik V Rodino-Klapac LR Viollet L Wall C King W Al-Dahhak R Gentamicin-induced readthrough of stop codons in duchenne muscular dystrophy. *Ann Neurol.* (2010) 67:771–80. 10.1002/ana.22024 20517938

[19] Kaufman RJ. Correction of genetic disease by making sense from nonsense. *J Clin Invest.* (1999) 104:367–8. 10.1172/JCI8055 10449426 PMC408532

[20] Sun C Shen L Zhang Z Xie X. Therapeutic strategies for duchenne muscular dystrophy: an update. *Genes (Basel).* (2020) 11:837. 10.3390/genes11080837 32717791 PMC7463903

[21] Barton-Davis ER Cordier L Shoturma DI Leland SE Sweeney HL. Aminoglycoside antibiotics restore dystrophin function to skeletal muscles of mdx mice. *J Clin Invest.* (1999) 104:375–81. 10.1172/JCI7866 10449429 PMC481050

[22] Wagner KR Hamed S Hadley DW Gropman AL Burstein AH Escolar DM Gentamicin treatment of duchenne and becker muscular dystrophy due to nonsense mutations. *Ann Neurol.* (2001) 49:706–11. 10.1002/ana.1023 11409421

[23] Politano L Nigro G Nigro V Piluso G Papparella S Paciello O Gentamicin administration in duchenne patients with premature stop codon. preliminary results. *Acta Myol.* (2003) 22:15–21. 12966700

[24] Hayward RS Harding J Molloy R Land L Longcroft-Neal K Moore D Adverse effects of a single dose of gentamicin in adults: a systematic review. *Br J Clin Pharmacol.* (2018) 84:223–38. 10.1111/bcp.13439 28940715 PMC5777443

[25] Dabrowski M Bukowy-Bieryllo Z Zietkiewicz E. Advances in therapeutic use of a drug-stimulated translational readthrough of premature termination codons. *Mol Med.* (2018) 24:25. 10.1186/s10020-018-0024-7 30134808 PMC6016875

[26] Mullard A. EMA reconsiders ‘read-through’ drug against duchenne muscular dystrophy following appeal. *Nat Biotechnol.* (2014) 32:706–706. 10.1038/nbt0814-706 25101726

[27] McDonald CM Campbell C Torricelli RE Finkel RS Flanigan KM Goemans N Ataluren in patients with nonsense mutation duchenne muscular dystrophy (ACT DMD): a multicentre, randomised, double-blind, placebo-controlled, phase 3 trial. *Lancet.* (2017) 390:1489–98. 10.1016/S0140-6736(17)31611-2 28728956

[28] Jesse’s Journey. *PTC Therapeutics: Hosts Call to Review Results of TranslarnaTM (ataluren) | Duchenne Muscular Dystrophy | Jesse’s Journey | Canada [Internet]. Duchenne Muscular Dystrophy | Jesse’s Journey.* (2021). Available online at: https://www.jessesjourney.com/ptc-therapeutics-hosts-call-to-review-results-of-translarna-ataluren/ (accessed May 30, 2021).

[29] Sardone V Zhou H Muntoni F Ferlini A Falzarano M. Antisense oligonucleotide-based therapy for neuromuscular disease. *Molecules.* (2017) 22:563. 10.3390/molecules22040563 28379182 PMC6154734

[30] Bennett CF Krainer AR Cleveland DW. Antisense oligonucleotide therapies for neurodegenerative diseases. *Annu Rev Neurosci.* (2019) 42:385–406. 10.1146/annurev-neuro-070918-050501 31283897 PMC7427431

[31] Bennett CF Swayze EE. RNA targeting therapeutics: molecular mechanisms of antisense oligonucleotides as a therapeutic platform. *Annu Rev Pharmacol Toxicol.* (2010) 50:259–93. 10.1146/annurev.pharmtox.010909.105654 20055705

[32] Fairclough RJ Wood MJ Davies KE. Therapy for duchenne muscular dystrophy: renewed optimism from genetic approaches. *Nat Rev Genet.* (2013) 14:373–8. 10.1038/nrg3460 23609411

[33] Guiraud S Chen H Burns DT Davies KE. Advances in genetic therapeutic strategies for duchenne muscular dystrophy. *Exp Physiol.* (2015) 100:1458–67. 10.1113/EP085308 26140505 PMC4973818

[34] Aartsma-Rus A Krieg AMFDA. Approves eteplirsen for duchenne muscular dystrophy: the next chapter in the eteplirsen saga. *Nucleic Acid Ther.* (2017) 27:1–3. 10.1089/nat.2016.0657 27929755 PMC5312460

[35] Komaki H Takeshima Y Matsumura T Ozasa S Funato M Takeshita E Viltolarsen in Japanese duchenne muscular dystrophy patients: a phase 1/2 study. *Ann Clin Transl Neurol.* (2020) 7:2393–408. 10.1002/acn3.51235 33285037 PMC7732240

[36] Frank DE Schnell FJ Akana C El-Husayni SH Desjardins CA Morgan J Increased dystrophin production with golodirsen in patients with duchenne muscular dystrophy. *Neurology.* (2020) 94:e2270–82. 10.1212/WNL.0000000000009233 32139505 PMC7357297

[37] FDA. *FDA Grants Accelerated Approval to First Drug for Duchenne Muscular Dystrophy [Internet].* (2020). Available online at: https://www. fda.gov/news-events/press-announcements/fda-grants-accelerated-approval-first-drug- duchenne-muscular-dystrophy (accessed June 8, 2021).

[38] Brogna C Coratti G Pane M Ricotti V Messina S D’Amico A Long-term natural history data in duchenne muscular dystrophy ambulant patients with mutations amenable to skip exons 44, 45, 51 and 53. *PLoS One.* (2019) 14:e0218683. 10.1371/journal.pone.0218683 31237898 PMC6592545

[39] Sarepta Therapeutics. *Sarepta Therapeutics Reports Positive Clinical Results from Phase 2 MOMENTUM Study of SRP-5051 in Patients with Duchenne Muscular Dystrophy Amenable to Skipping Exon 51 | Sarepta Therapeutics, Inc. [Internet].* (2021). Available online at: https://investorrelations.sarepta.com/news-releases/news-release-details/sarepta-therapeutics-reports- positive-clinical-results-phase-2 (accessed August 26, 2021).

[40] Clemens PR Rao VK Connolly AM Harper AD Mah JK Smith EC Safety, tolerability, and efficacy of viltolarsen in boys with duchenne muscular dystrophy amenable to exon 53 skipping: a phase 2 randomized clinical trial. *JAMA Neurol.* (2020) 77:982. 10.1001/jamaneurol.2020.1264 32453377 PMC7251505

[41] Sarepta Therapeutics. *Sarepta Therapeutics Announces FDA Approval of AMONDYS 45TM (casimersen) Injection for the Treatment of Duchenne Muscular Dystrophy (DMD) in Patients Amenable to Skipping Exon 45 | Sarepta Therapeutics, Inc. [Internet].* (2021). Available online at: https://investorrelations. sarepta.com/news-releases/news-release-details/sarepta-therapeutics- announces-fda-approval-amondys-45tm (accessed June 8, 2021).

[42] Muscular Dystrophy. *Amondys 45 (Casimersen) - Muscular Dystrophy News [Internet].* (2021). Available online at: https://musculardystrophynews.com/srp-4045/ (accessed June 8, 2021).

[43] Kolev NG Steitz JA. In vivo assembly of functional U7 snRNP requires RNA backbone flexibility within the Sm-binding site. *Nat Struct Mol Biol.* (2006) 13:347–53. 10.1038/nsmb1075 16547514

[44] Will CL Lührmann R. Spliceosome structure and function. *Cold Spring Harb Perspect Biol.* (2011) 3:a003707. 10.1101/cshperspect.a003707 21441581 PMC3119917

[45] Cazzella V Martone J Pinnarò C Santini T Twayana SS Sthandier O Exon 45 skipping through U1-snRNA antisense molecules recovers the Dys-nNOS pathway and muscle differentiation in human DMD myoblasts. *Mol Ther.* (2012) 20:2134–42. 10.1038/mt.2012.178 22968481 PMC3498801

[46] Goyenvalle A Vulin A Fougerousse F Leturcq F Kaplan J-C Garcia L Rescue of dystrophic muscle through U7 snRNA-mediated exon skipping. *Science.* (2004) 306:1796–9. 10.1126/science.1104297 15528407

[47] Goyenvalle A Wright J Babbs A Wilkins V Garcia L Davies KE. Engineering multiple U7snRNA constructs to induce single and multiexon-skipping for duchenne muscular dystrophy. *Mol Ther.* (2012) 20:1212–21. 10.1038/mt.2012.26 22354379 PMC3369406

[48] Vulin A Wein N Simmons TR Rutherford AM Findlay AR Yurkoski JA The first exon duplication mouse model of duchenne muscular dystrophy: a tool for therapeutic development. *Neuromuscul Disord.* (2015) 25:827–34. 10.1016/j.nmd.2015.08.005 26365037

[49] Simmons TR Vetter TA Huang N Vulin-Chaffiol A Wein N Flanigan KM. Pre-clinical dose-escalation studies establish a therapeutic range for U7snRNA-mediated DMD exon 2 skipping. *Mol Ther Methods Clin Dev.* (2021) 21:325–40. 10.1016/j.omtm.2021.03.014 33898631 PMC8047432

[50] Gushchina LV Frair EC Rohan N Bradley AJ Simmons TR Chavan HD Lack of toxicity in nonhuman primates receiving clinically relevant doses of an AAV9.U7snRNA vector designed to induce DMD exon 2 skipping. *Hum Gene Ther.* (2021) 32:882–94. 10.1089/hum.2020.286 33406986 PMC10112461

[51] Flanigan K Wein N Gushchina L Waldrop M Weiss RP. 140RNA-Seq shows an absence of off-target splicing effects in AAV9-U7snRNA mediated skipping of DMD exon 2. *Neuromuscul Disord.* (2019) 29:S89.

[52] Wein N Dunn DM Waldrop MA Gushchina LV Frair EC Weiss RB Absence of significant off-target splicing variation with a U7snRNA vector targeting DMD exon 2 duplications. *Hum Gene Ther.* (2021) 32:1346–59. 10.1089/hum.2020.315 34060935

[53] Rodríguez-Rodríguez DR Ramírez-Solís R Garza-Elizondo MA De Lourdes Garza-Rodríguez M Barrera-Saldaña HA. Genome editing: a perspective on the application of CRISPR/Cas9 to study human diseases (review). *Int J Mol Med.* (2019) 43:1559–74. 10.3892/ijmm.2019.4112 30816503 PMC6414166

[54] Wang H La Russa M Qi LS. CRISPR/Cas9 in genome editing and beyond. *Annu Rev Biochem.* (2016) 85:227–64. 27145843 10.1146/annurev-biochem-060815-014607 PMC13384723

[55] Jiang F Doudna JA. CRISPR–Cas9 structures and mechanisms. *Annu Rev Biophys.* (2017) 46:505–29. 10.1146/annurev-biophys-062215-010822 28375731

[56] Porto EM Komor AC Slaymaker IM Yeo GW. Base editing: advances and therapeutic opportunities. *Nat Rev Drug Discov.* (2020) 19:839–59. 10.1038/s41573-020-0084-6 33077937 PMC7721651

[57] Anzalone AV Randolph PB Davis JR Sousa AA Koblan LW Levy JM Search-and-replace genome editing without double-strand breaks or donor DNA. *Nature.* (2019) 576:149–57. 10.1038/s41586-019-1711-4 31634902 PMC6907074

[58] Happi Mbakam C Lamothe G Tremblay G Tremblay JP. CRISPR-Cas9 gene therapy for duchenne muscular dystrophy. *Neurotherapeutics [Internet].* (2022). 10.1007/s13311-022-01197-9 [Epub ahead of print]. 35165856 PMC9294086

[59] Iyombe-Engembe J-P Ouellet DL Barbeau X Rousseau J Chapdelaine P Lagüe P Efficient restoration of the dystrophin gene reading frame and protein structure in DMD myoblasts using the CinDel method. *Mol Ther Nucleic Acids.* (2016) 5:e283. 10.1038/mtna.2015.58 26812655 PMC5012554

[60] Duchêne BL Cherif K Iyombe-Engembe J-P Guyon A Rousseau J Ouellet DL CRISPR-induced deletion with sacas9 restores dystrophin expression in dystrophic models in vitro and in vivo. *Mol Ther.* (2018) 26:2604–16. 10.1016/j.ymthe.2018.08.010 30195724 PMC6224775

[61] Xu L Park KH Zhao L Xu J El Refaey M Gao Y CRISPR-mediated genome editing restores dystrophin expression and function in mdx mice. *Mol Ther.* (2016) 24:564–9. 10.1038/mt.2015.192 26449883 PMC4786912

[62] Koo T Lu-Nguyen NB Malerba A Kim E Kim D Cappellari O Functional rescue of dystrophin deficiency in mice caused by frameshift mutations using campylobacter jejuni Cas9. *Mol Ther.* (2018) 26:1529–38. 10.1016/j.ymthe.2018.03.018 29730196 PMC5986736

[63] Min Y-L Li H Rodriguez-Caycedo C Mireault AA Huang J Shelton JM CRISPR-Cas9 corrects duchenne muscular dystrophy exon 44 deletion mutations in mice and human cells. *Sci Adv.* (2019) 5:eaav4324. 10.1126/sciadv.aav4324 30854433 PMC6402849

[64] Domenig SA Bundschuh N Lenardič A Ghosh A Kim I Qabrati X CRISPR/Cas9 editing of directly reprogrammed myogenic progenitors restores dystrophin expression in a mouse model of muscular dystrophy. *Stem Cell Rep.* (2022) 17:321–36. 10.1016/j.stemcr.2021.12.003 34995499 PMC8828535

[65] Rees HA Liu DR. Base editing: precision chemistry on the genome and transcriptome of living cells. *Nat Rev Genet.* (2018) 19:770–88. 10.1038/s41576-018-0059-1 30323312 PMC6535181

[66] Xu L Zhang C Li H Wang P Gao Y Mokadam NA Efficient precise in vivo base editing in adult dystrophic mice. *Nat Commun.* (2021) 12:3719. 10.1038/s41467-021-23996-y 34140489 PMC8211797

[67] Zuo E Sun Y Wei W Yuan T Ying W Sun H Cytosine base editor generates substantial off-target single-nucleotide variants in mouse embryos. *Science.* (2019) 364:289–92. 10.1126/science.aav9973 30819928 PMC7301308

[68] Chemello F Chai AC Li H Rodriguez-Caycedo C Sanchez-Ortiz E Atmanli A Precise correction of duchenne muscular dystrophy exon deletion mutations by base and prime editing. *Sci Adv.* (2021) 7:eabg4910. 10.1126/sciadv.abg4910 33931459 PMC8087404

[69] Liu P Liang S-Q Zheng C Mintzer E Zhao YG Ponnienselvan K Improved prime editors enable pathogenic allele correction and cancer modelling in adult mice. *Nat Commun.* (2021) 12:2121. 10.1038/s41467-021-22295-w 33837189 PMC8035190

[70] Chen PJ Hussmann JA Yan J Knipping F Ravisankar P Chen P-F Enhanced prime editing systems by manipulating cellular determinants of editing outcomes. *Cell.* (2021) 184:5635.e–52.e. 10.1016/j.cell.2021.09.018 34653350 PMC8584034

[71] Tremblay G Rousseau J Mbakam CH Tremblay JP. Insertion of the icelandic mutation (A673T) by prime editing: a potential preventive treatment for familial and sporadic alzheimer’s disease. *CRISPR J.* (2022) 5:109–22. 10.1089/crispr.2021.0085 35133877 PMC9009594

[72] Rousseau J Mbakam CH Guyon A Tremblay G Begin FG Tremblay JP. Specific mutations in genes responsible for Alzheimer and for duchenne muscular dystrophy introduced by base editing and PRIME editing. *bioRxiv.* (2020): 10.1101/2020.07.31.230565

[73] Consalvi S Saccone V Mozzetta C. Histone deacetylase inhibitors: a potential epigenetic treatment for c muscular dystrophy. *Epigenomics.* (2014) 6:547–60. 10.2217/epi.14.36 25431946

[74] Yin H Price F Rudnicki MA. Satellite cells and the muscle stem cell niche. *Physiol Rev.* (2013) 93:23–67. 10.1152/physrev.00043.2011 23303905 PMC4073943

[75] Judson RN Zhang R-H Rossi FMA. Tissue-resident mesenchymal stem/progenitor cells in skeletal muscle: collaborators or saboteurs? *FEBS J.* (2013) 280:4100–8. 10.1111/febs.12370 23763717 PMC4880469

[76] Dellavalle A Sampaolesi M Tonlorenzi R Tagliafico E Sacchetti B Perani L Pericytes of human skeletal muscle are myogenic precursors distinct from satellite cells. *Nat Cell Biology.* (2007) 9:255–67. 10.1038/ncb1542 17293855

[77] Asakura A Seale P Girgis-Gabardo A Rudnicki MA. Myogenic specification of side population cells in skeletal muscle. *J Cell Biol.* (2002) 159:123–34. 10.1083/jcb.200202092 12379804 PMC2173497

[78] Gussoni E Soneoka Y Strickland CD Buzney EA Khan MK Flint AF Dystrophin expression in the mdx mouse restored by stem cell transplantation. *Nature.* (1999) 401:390–4. 10.1038/43919 10517639

[79] Mitchell KJ Pannérec A Cadot B Parlakian A Besson V Gomes ER Identification and characterization of a non-satellite cell muscle resident progenitor during postnatal development. *Nat Cell Biology.* (2010) 12:257–66. 10.1038/ncb2025 20118923

[80] Minetti GC Colussi C Adami R Serra C Mozzetta C Parente V Functional and morphological recovery of dystrophic muscles in mice treated with deacetylase inhibitors. *Nat Med.* (2006) 12:1147–50. 10.1038/nm1479 16980968

[81] Vojinovic J Damjanov N D’Urzo C Furlan A Susic G Pasic S Safety and efficacy of an oral histone deacetylase inhibitor in systemic-onset juvenile idiopathic arthritis. *Arthritis Rheum.* (2011) 63:1452–8. 10.1002/art.30238 21538322

[82] Bettica P Petrini S D’Oria V D’Amico A Catteruccia M Pane M Histological effects of givinostat in boys with duchenne muscular dystrophy. *Neuromuscul Disord.* (2016) 26:643–9. 10.1016/j.nmd.2016.07.002 27566866

[83] businesswire. *Italfarmaco Provides Update on Ongoing Clinical Programs with Givinostat in Oral Presentation at XVIII International Conference on Duchenne and Becker Muscular Dystrophy [Internet].* (2021). Available online at: https://www.businesswire.com/news/home/20210222005359/en/Italfarmaco-Provides-Update-on-Ongoing-Clinical-Programs-with-Givinostat-in-Oral-Presentation-at-XVIII-International-Conference-on-Duchenne-and-Becker-Muscular-Dystrophy (accessed February 18, 2022).

[84] European Medicines Agency. *EU/3/12/1009: Orphan Designation for the Treatment of Duchenne Muscular Dystrophy [Internet].* (2018). Available online at: https://www.ema.europa.eu/en/medicines/human/orphan-designations/eu3121009 (accessed February 4, 2021).

[85] Piovesan A Caracausi M Antonaros F Pelleri MC Vitale L. GeneBase 1.1: a tool to summarize data from NCBI gene datasets and its application to an update of human gene statistics. *Database.* (2016) 2016:baw153. 10.1093/database/baw153 28025344 PMC5199132

[86] Benedetti S Uno N Hoshiya H Ragazzi M Ferrari G Kazuki Y Reversible immortalisation enables genetic correction of human muscle progenitors and engineering of next-generation human artificial chromosomes for duchenne muscular dystrophy. *EMBO Mol Med.* (2018) 10:254–75. 10.15252/emmm.201607284 29242210 PMC5801502

[87] Meng J Sweeney NP Doreste B Muntoni F McClure M Morgan J. Restoration of functional full-length dystrophin after intramuscular transplantation of foamy virus-transduced myoblasts. *Hum Gene Ther.* (2020) 31:241–52. 10.1089/hum.2019.224 31801386 PMC7047098

[88] Duan D. Systemic AAV micro-dystrophin gene therapy for duchenne muscular dystrophy. *Mol Ther.* (2018) 26:2337–56. 10.1016/j.ymthe.2018.07.011 30093306 PMC6171037

[89] Harper SQ Hauser MA DelloRusso C Duan D Crawford RW Phelps SF Modular flexibility of dystrophin: implications for gene therapy of duchenne muscular dystrophy. *Nat Med.* (2002) 8:253–61. 10.1038/nm0302-253 11875496

[90] Wang B Li J Xiao X. Adeno-associated virus vector carrying human minidystrophin genes effectively ameliorates muscular dystrophy in mdx mouse model. *Proc Natl Acad Sci USA.* (2000) 97:13714–9. 10.1073/pnas.240335297 11095710 PMC17641

[91] Hakim CH Wasala NB Pan X Kodippili K Yue Y Zhang K A five-repeat micro-dystrophin gene ameliorated dystrophic phenotype in the severe dba/2j-mdx model of duchenne muscular dystrophy. *Mol Ther Methods Clin Dev.* (2017) 6:216–30. 10.1016/j.omtm.2017.06.006 28932757 PMC5596503

[92] Tandon A Jefferies JL Villa CR Hor KN Wong BL Ware SM Dystrophin genotype–cardiac phenotype correlations in duchenne and becker muscular dystrophies using cardiac magnetic resonance imaging. *Am J Cardiol.* (2015) 115:967–71. 10.1016/j.amjcard.2015.01.030 25702278 PMC5568575

[93] Wang H Marrosu E Brayson D Wasala NB Johnson EK Scott CS Proteomic analysis identifies key differences in the cardiac interactomes of dystrophin and micro-dystrophin. *Hum Mol Genet.* (2021) 30:1321–36. 10.1093/hmg/ddab133 33949649 PMC8255133

[94] Bourdon A François V Zhang L Lafoux A Fraysse B Toumaniantz G Evaluation of the dystrophin carboxy-terminal domain for micro-dystrophin gene therapy in cardiac and skeletal muscles in the DMDmdx rat model. *Gene Ther.* (2022) 1:1–16. 10.1038/s41434-022-00317-6 35105949

[95] Mendell JR Sahenk Z Lehman K Nease C Lowes LP Miller NF Assessment of systemic delivery of rAAVrh74.MHCK7.micro-dystrophin in children with duchenne muscular dystrophy: a nonrandomized controlled trial. *JAMA Neurol.* (2020) 77:1122. 10.1001/jamaneurol.2020.1484 32539076 PMC7296461

[96] Willcocks RJ Forbes SC Walter GA Sweeney L Rodino-Klapac LR Mendell JR Assessment of rAAVrh.74.MHCK7.micro-dystrophin gene therapy using magnetic resonance imaging in children with duchenne muscular dystrophy. *JAMA Netw Open.* (2021) 4:e2031851. 10.1001/jamanetworkopen.2020.31851 33394000 PMC7783546

[97] Srivastava A. In vivo tissue-tropism of adeno-associated viral vectors. *Curr Opin Virol.* (2016) 21:75–80. 10.1016/j.coviro.2016.08.003 27596608 PMC5138125

[98] Philippidis A. After patient death, FDA places hold on pfizer duchenne muscular dystrophy gene therapy trial. *Hum Gene Ther.* (2022) 33:111–5. 10.1089/hum.2022.29198.bfs 35696528

[99] PFIZER. *Pfizer Presents Initial Clinical Data on Phase 1b Gene Therapy Study for Duchenne Muscular Dystrophy (DMD) | Pfizer [Internet].* (2019). Available online at: https://www.pfizer.com/news/press-release/pressdetail/pfizer_presents_initial_clinical_data_on_phase_1b_gene_therapy_study_for_duchenne_muscular_dystrophy_dmd (accessed February 17, 2022).

[100] Solid Biosciences. *Solid Biosciences Reports 1.5-Year Data from Patients in the Ongoing IGNITE DMD Phase I/II Clinical Trial of SGT-001 [Internet].* (2021). Available online at: https://www.solidbio.com/about/media/press-releases/solid-biosciences-reports-1-5-year-data-from-patients-in-the-ongoing-ignite-dmd-phase-i-ii-clinical-trial-of-sgt-001 (accessed February 18, 2022).

[101] Sun C Serra C Lee G Wagner K. Stem cell-based therapies for duchenne muscular dystrophy. *Exp Neurol.* (2020) 323:113086. 10.1016/j.expneurol.2019.113086 31639376 PMC6899334

[102] Mueller AL Bloch RJ. Skeletal muscle cell transplantation: models and methods. *J Muscle Res Cell Motil.* (2020) 41:297–311. 10.1007/s10974-019-09550-w 31392564

[103] Riederer I Negroni E Bencze M Wolff A Aamiri A Di Santo JP Slowing down differentiation of engrafted human myoblasts into immunodeficient mice correlates with increased proliferation and migration. *Mol Ther.* (2012) 20:146–54. 10.1038/mt.2011.193 21934656 PMC3255588

[104] Skuk D Tremblay JP. First study of intra-arterial delivery of myogenic mononuclear cells to skeletal muscles in primates. *Cell Transplant.* (2014) 23(Suppl. 1):141–50. 10.3727/096368914X685032 25303080

[105] Skuk D Goulet M Roy B Piette V Côté CH Chapdelaine P First test of a “high-density injection” protocol for myogenic cell transplantation throughout large volumes of muscles in a duchenne muscular dystrophy patient: eighteen months follow-up. *Neuromuscul Disord.* (2007) 17:38–46. 10.1016/j.nmd.2006.10.003 17142039

[106] Skuk D Tremblay JP. Cell therapy in myology: dynamics of muscle precursor cell death after intramuscular administration in non-human primates. *Mol Ther Methods Clin Dev.* (2017) 5:232–40. 10.1016/j.omtm.2017.05.002 28573152 PMC5447384

[107] Maffioletti SM Noviello M English K Tedesco FS. Stem cell transplantation for muscular dystrophy: the challenge of immune response. *Biomed Res Int.* (2014) 2014:964010. 10.1155/2014/964010 25054157 PMC4098613

[108] Skuk D Goulet M Roy B Chapdelaine P Bouchard J-P Roy R Dystrophin expression in muscles of duchenne muscular dystrophy patients after high-density injections of normal myogenic cells. *J Neuropathol Exp Neurol.* (2006) 65:371–86. 10.1097/01.jnen.0000218443.45782.81 16691118

[109] Skuk D Roy B Goulet M Chapdelaine P Bouchard J-P Roy R Dystrophin expression in myofibers of duchenne muscular dystrophy patients following intramuscular injections of normal myogenic cells. *Mol Ther.* (2004) 9:475–82. 10.1016/j.ymthe.2003.11.023 15038390

[110] Rajput BS Chakrabarti SK Dongare VS Ramirez CM Deb KD. Human umbilical cord mesenchymal stem cells in the treatment of duchenne muscular dystrophy: safety and feasibility study in india. *J Stem Cells.* (2015) 10:141–56. 27125141

[111] Blake DJ Tinsley JM Davies KE. Utrophin: a structural and functional comparison to dystrophin. *Brain Pathol.* (1996) 6:37–47. 10.1111/j.1750-3639.1996.tb00781.x 8866746

[112] Mattei E Corbi N Di Certo MG Strimpakos G Severini C Onori A Utrophin up-regulation by an artificial transcription factor in transgenic mice. *PLoS One.* (2007) 2:e774. 10.1371/journal.pone.0000774 17712422 PMC1942121

[113] Pisani C Strimpakos G Gabanella F Di Certo MG Onori A Severini C Utrophin up-regulation by artificial transcription factors induces muscle rescue and impacts the neuromuscular junction in mdx mice. *Biochim Biophys Acta Mol Basis Dis.* (2018) 1864:1172–82. 10.1016/j.bbadis.2018.01.030 29408646 PMC5851675

[114] Loro E Sengupta K Bogdanovich S Whig K Schultz DC Huryn DM High-throughput identification of post-transcriptional utrophin up-regulators for duchenne muscle dystrophy (DMD) therapy. *Sci Rep.* (2020) 10:2132. 10.1038/s41598-020-58737-6 PMC7005813 32034254

[115] Sengupta K Mishra MK Loro E Spencer MJ Pyle AD Khurana TS. Genome editing-mediated utrophin upregulation in duchenne muscular dystrophy stem cells. *Mol Ther Nucleic Acids.* (2020) 22:500–9. 10.1016/j.omtn.2020.08.031 33230452 PMC7554652

[116] Péladeau C Adam N Bronicki LM Coriati A Thabet M Al-Rewashdy H Identification of therapeutics that target eEF1A2 and upregulate utrophin a translation in dystrophic muscles. *Nat Commun.* (2020) 11:1990. 10.1038/s41467-020-15971-w 32332749 PMC7181625

[117] Muntoni F Tejura B Spinty S Roper H Hughes I Layton G a phase 1b trial to assess the pharmacokinetics of ezutromid in pediatric duchenne muscular dystrophy patients on a balanced diet. *Clin Pharmacol Drug Dev.* (2019) 8:922–33. 10.1002/cpdd.642 30650257

[118] Muntoni F Maresh K Davies K Harriman S Layton G Rosskamp R PhaseOut DMD: a phase 2, proof of concept, clinical study of utrophin modulation with ezutromid. *Neuromuscul Disord.* (2017) 27: S217.

[119] Jason TLH Koropatnick J Berg RW. Toxicology of antisense therapeutics. *Toxicol Appl Pharmacol.* (2004) 201:66–83. 10.1016/j.taap.2004.04.017 15519609

[120] Li N Parkes JE Spathis R Morales M Mcdonald J Kendra RM The effect of immunomodulatory treatments on anti-dystrophin immune response after aav gene therapy in dystrophin deficient mdx mice. *J Neuromuscul Dis.* (2021) 8:S325–40. 10.3233/JND-210706 34569971

[121] Le Guiner C Servais L Montus M Larcher T Fraysse B Moullec S Long-term microdystrophin gene therapy is effective in a canine model of duchenne muscular dystrophy. *Nat Commun.* (2017) 8:16105. 10.1038/ncomms16105 28742067 PMC5537486

[122] Chicoine LG Montgomery CL Bremer WG Shontz KM Griffin DA Heller KN Plasmapheresis eliminates the negative impact of AAV antibodies on microdystrophin gene expression following vascular delivery. *Mol Ther.* (2014) 22:338–47. 10.1038/mt.2013.244 24196577 PMC3916040

[123] Velazquez VM Meadows AS Pineda RJ Camboni M McCarty DM Fu H. Effective depletion of pre-existing anti-aav antibodies requires broad immune targeting. *Mol Ther Methods Clin Dev.* (2017) 4:159–68. 10.1016/j.omtm.2017.01.003 28345001 PMC5363314

[124] Mendell JR Campbell K Rodino-Klapac L Sahenk Z Shilling C Lewis S Dystrophin immunity in duchenne’s muscular dystrophy. *N Engl J Med.* (2010) 363:1429–37. 10.1056/NEJMoa1000228 20925545 PMC3014106

[125] FDA. *Cellular, Tissue, and Gene Therapies Advisory Committee September 2-3, 2021 Meeting Announcement - 09/02/2021 - 09/03/2021 [Internet].* (2021). Available online at: https://www.fda.gov/advisory-committees/advisory-committee-calendar/cellular-tissue-and-gene-therapies-advisory-committee-september-2-3-2021-meeting-announcement (accessed February 15, 2022).

[126] Philippidis A. After third death, audentes’ AT132 remains on clinical hold. *Hum Gene Ther.* (2020) 31:908–10. 10.1089/hum.2020.29133.bfs 32945722

[127] Philippidis A. Fourth boy dies in clinical trial of astellas’ AT132. *Hum Gene Ther.* (2021) 32:1008–10. 10.1089/hum.2021.29182.bfs 34662231

[128] Dalwadi DA Calabria A Tiyaboonchai A Posey J Naugler WE Montini E AAV integration in human hepatocytes. *Mol Ther.* (2021) 29:2898–909. 10.1016/j.ymthe.2021.08.031 34461297 PMC8531150

[129] Xu CL Ruan MZC Mahajan VB Tsang SH. Viral delivery systems for CRISPR. *Viruses.* (2019) 11:28. 10.3390/v11010028 30621179 PMC6356701

[130] Li X Corbett AL Taatizadeh E Tasnim N Little JP Garnis C Challenges and opportunities in exosome research—Perspectives from biology, engineering, and cancer therapy. *APL Bioeng.* (2019) 3:011503. 10.1063/1.5087122 31069333 PMC6481742

[131] Ramamoorth M Narvekar A. Non viral vectors in gene therapy- an overview. *J Clin Diagn Res.* (2015) 9:GE01–6. 10.7860/JCDR/2015/10443.5394 25738007 PMC4347098

[132] Wu Z Asokan A Samulski RJ. Adeno-associated virus serotypes: vector toolkit for human gene therapy. *Mol Ther.* (2006) 14:316–27. 10.1016/j.ymthe.2006.05.009 16824801

[133] Koo T Popplewell L Athanasopoulos T Dickson G. Triple trans-splicing adeno-associated virus vectors capable of transferring the coding sequence for full-length dystrophin protein into dystrophic mice. *Hum Gene Ther.* (2014) 25:98–108. 10.1089/hum.2013.164 24191945

[134] Reisinger E. Dual-AAV delivery of large gene sequences to the inner ear. *Hear Res.* (2020) 394:107857. 10.1016/j.heares.2019.107857 31810595

[135] Tornabene P Trapani I Minopoli R Centrulo M Lupo M de Simone S Intein-mediated protein trans-splicing expands adeno-associated virus transfer capacity in the retina. *Sci Transl Med.* (2019) 11:eaav4523. 10.1126/scitranslmed.aav4523 31092694 PMC6863751

[136] Bobis-Wozowicz S Kania K Nit K Blazowska N Kmiotek-Wasylewska K Paw M Efficient in vivo genome editing mediated by stem cells-derived extracellular vesicles carrying designer nucleases. *bioRxiv.* (2021). 10.1101/2021.02.25.432823

[137] Gee P Lung MSY Okuzaki Y Sasakawa N Iguchi T Makita Y Extracellular nanovesicles for packaging of CRISPR-Cas9 protein and sgRNA to induce therapeutic exon skipping. *Nat Commun.* (2020) 11:1334. 10.1038/s41467-020-14957-y 32170079 PMC7070030

[138] Scoles DR Minikel EV Pulst SM. Antisense oligonucleotides. *Neurol Genet.* (2019) 5:e323. 10.1212/NXG.0000000000000323 PMC6501637 31119194

[139] Schnepp BC Clark KR Klemanski DL Pacak CA Johnson PR. Genetic fate of recombinant adeno-associated virus vector genomes in muscle. *J Virol.* (2003) 77:3495–504. 10.1128/jvi.77.6.3495-3504.2003 12610125 PMC149530

[140] Nakai H Yant SR Storm TA Fuess S Meuse L Kay MA. Extrachromosomal recombinant adeno-associated virus vector genomes are primarily responsible for stable liver transduction in vivo. *J Virol.* (2001) 75:6969–76. 10.1128/JVI.75.15.6969-6976.2001 11435577 PMC114425

[141] Hagedorn C Schnödt-Fuchs M Boehme P Abdelrazik H Lipps HJ Büning HS. /MAR element facilitates episomal long-term persistence of adeno-associated virus vector genomes in proliferating cells. *Hum Gene Ther.* (2017) 28:1169–79. 10.1089/hum.2017.025 28665147

[142] Zhang W Solanki M Müther N Ebel M Wang J Sun C Hybrid adeno-associated viral vectors utilizing transposase-mediated somatic integration for stable transgene expression in human cells. *PLoS One.* (2020) 15:e0228707. 10.1371/journal.pone.0228707 31999798 PMC6992166

[143] Howard ZM Dorn LE Lowe J Gertzen MD Ciccone P Rastogi N Micro-dystrophin gene therapy prevents heart failure in an improved Duchenne muscular dystrophy cardiomyopathy mouse model. *JCI Insight.* (2021) 6:e146511. 10.1172/jci.insight.146511 33651713 PMC8119181

[144] Walton RT Christie KA Whittaker MN Kleinstiver BP. Unconstrained genome targeting with near-PAMless engineered CRISPR-Cas9 variants. *Science.* (2020) 368:290–6. 10.1126/science.aba8853 32217751 PMC7297043

[145] Nelson JW Randolph PB Shen SP Everette KA Chen PJ Anzalone AV Engineered pegRNAs improve prime editing efficiency. *Nat Biotechnol.* (2021) 4:1–9. 10.1038/s41587-021-01039-7 PMC8930418 34608327

[146] Li C Samulski RJ. Engineering adeno-associated virus vectors for gene therapy. *Nat Rev Genet.* (2020) 21:255–72. 32042148 10.1038/s41576-019-0205-4

[147] Duan D Goemans N Takeda S Mercuri E Aartsma-Rus A. Duchenne muscular dystrophy. *Nat Rev Dis Primers.* (2021) 7:1–19. 10.1093/med/9780199681488.003.0001 33602943 PMC10557455

[148] Olson EN. Toward the correction of muscular dystrophy by gene editing. *Proc Natl Acad Sci USA.* (2021) 118:e2004840117. 10.1073/pnas.2004840117 34074727 PMC8179164

[149] Yuasa K Ishii A Miyagoe Y Takeda S. [Introduction of rod-deleted dystrophin cDNA, delta DysM3, into mdx skeletal muscle using adenovirus vector]. *Nihon Rinsho.* (1997) 55: 3148–53. 9436426

[150] Saccone V Consalvi S Giordani L Mozzetta C Barozzi I Sandoná M HDAC-regulated myomiRs control BAF60 variant exchange and direct the functional phenotype of fibro-adipogenic progenitors in dystrophic muscles. *Genes Dev.* (2014) 28:841–57. 10.1101/gad.234468.113 24682306 PMC4003277

[151] Tedesco FS Hoshiya H D’Antona G Gerli MFM Messina G Antonini S Stem cell–mediated transfer of a human artificial chromosome ameliorates muscular dystrophy. *Sci Transl Med.* (2011) 3:96ra78. 10.1126/scitranslmed.3002342 21849666

